# Disentangling the Microphysical Effects of Fire Particles on Convective Clouds Through A Case Study

**DOI:** 10.1029/2019JD031890

**Published:** 2020-06-16

**Authors:** Azusa Takeishi, Trude Storelvmo, Laura Fierce

**Affiliations:** ^1^ Department of Geology and Geophysics Yale University New Haven CT USA; ^2^ Currently at Laboratoire d'Aérologie University of Toulouse/CNRS Toulouse France; ^3^ Department of Geosciences University of Oslo Oslo Norway; ^4^ Environmental and Climate Sciences Department Brookhaven National Laboratory Upton NY USA

**Keywords:** aerosol‐cloud interaction, aerosol activation, WRF‐CHEM

## Abstract

Aerosol emissions from forest fires may impact cloud droplet activation through an increase in particle number concentrations (“the number effect”) and also through a decrease in the hygroscopicity * κ* of the entire aerosol population (“the hygroscopicity effect”) when fully internal mixing is assumed in models. This study investigated these effects of fire particles on the properties of simulated deep convective clouds (DCCs), using cloud‐resolving simulations with the Weather Research and Forecasting model coupled with Chemistry for a case study in a partly idealized setting. We found that the magnitude of the hygroscopicity effect was in some cases strong enough to entirely offset the number/size effect, in terms of its influence on modeled droplet and ice crystal concentrations. More specifically, in the case studied here, the droplet number concentration was reduced by about 37% or more due solely to the hygroscopicity effect. In the atmosphere, by contrast, fire particles likely have a much weaker impact on the hygroscopicity of the pre‐existing background aerosol, as such a strong impact would occur only if the fire particles mixed immediately and uniformly with the background. We also show that the differences in the number of activated droplets eventually led to differences in the optical thickness of the clouds aloft, though this finding is limited to only a few hours of the initial development stage of the DCCs. These results suggest that accurately and rigorously representing aerosol mixing and * κ* in models is an important step toward accurately simulating aerosol‐cloud interactions under the influence of fires.

## Key Points

- Fire emissions increase aerosol loading and decrease the modeled aerosol hygroscopicity: These have opposite impacts on the simulated clouds
- The model assumption of fully internal aerosol mixing likely exaggerates the hygroscopicity change due to fire emissions
- The overestimation of the hygroscopicity effect may lead to underestimation of droplet numbers, especially in fire‐influenced environments

## 1 Introduction

Obtaining a deeper understanding of aerosol‐cloud interactions has been one of the crucial aims of weather and climate studies in recent years. Through their ability to serve as cloud condensation nuclei (CCN) and/or ice nucleating particles (INPs), aerosol particles may affect cloud properties, which can in turn affect atmospheric dynamics and the Earth's radiation budget on a variety of scales. The present study focuses on the impacts of particles from forest fires on deep convective clouds (DCCs), and, in particular, how they are simulated in a cloud‐resolving model.

For context, the following subsections briefly review some related studies on the impacts of aerosols on DCCs (1.1) and studies with a particular focus on the impacts of forest fire particles on DCCs (1.2).

### 1.1 Aerosol Indirect Effects on DCCs via the CCN Effects

DCCs are commonly observed in the tropics and summertime mid‐latitudes and play a crucial role in weather and climate through their vertical transport of heat, moisture, gases, and aerosols, as well as via their anvil clouds and intense precipitation. According to the Tropical Rainfall Measuring Mission (TRMM) satellite data analysis by Schumacher and Houze (2003), for instance, approximately 60% and 50% of the total precipitation in the tropics (20°S to 20°N) and the low/mid‐latitudes (25–35°S and 25–35°N) are of convective nature, respectively (see their Figure 2). Furthermore, changes in DCC anvil properties are expected to lead to substantial climate forcings and/or feedbacks (Hartmann, 2016; Koren et al., 2010). If any increase or decrease in aerosol concentrations affects DCCs and hence their anvil/precipitation nature (e.g., Saleeby et al., 2016), it would also likely impact the climate.

Earlier observational (satellite/aircraft) studies showed a delay in the onset of precipitation in DCCs concurrently with high aerosol concentrations (Andreae et al., 2004; Rosenfeld, 1999; Rosenfeld & Woodley, 2000). This eventually led to the establishment of a theory related to the thermodynamic effect of aerosols on DCCs, in which invigoration of DCCs occurs due to less efficient warm‐rain production, increased freezing of smaller and more numerous cloud droplets, and a resultant increase in latent heat release aloft (Altaratz et al., 2014; Andreae et al., 2004; Khain et al., 2005; Rosenfeld et al., 2008; van den Heever et al., 2006). This effect is now widely recognized and supported by observations (e.g., Andreae et al., 2004; Niu & Li, 2012) and theoretical studies (Koren et al., 2008; Rosenfeld et al., 2008). Furthermore, Sheffield et al. (2015) showed that the invigoration may also occur in the warm phase of DCCs through increased droplet surface areas and hence increased depositional growth. However, not all DCCs exhibit the invigoration effect of aerosols, as its occurrence heavily depends on environmental conditions such as instability represented by CAPE (Lee et al., 2008; Storer et al., 2010), humidity distribution (Fan et al., 2010; Grant & van den Heever, 2015), vertical wind shear (Fan et al., 2009; Lebo & Morrison, 2014), existence of a warm cloud base (Altaratz et al., 2014; Rosenfeld et al., 2008, 2014), and land surface properties such as soil moisture (Grant & van den Heever, 2014). This dependence on environmental characteristics and the wide variety of microphysical and dynamical conditions in which DCCs form make it challenging to fully understand aerosol‐DCC interactions. A number of modeling studies have also been focusing on aerosol‐DCC relationship in the last couple of decades (e.g., Khain et al., 2004; Phillips et al., 2002; Wang, 2005); some studies focused on a specific type of DCCs such as supercells (Khain & Lynn, 2009; Lebo et al., 2012; Lerach et al., 2008; Lim et al., 2011; Morrison, 2012), squall lines (Lebo & Morrison, 2014; Li et al., 2009; Seigel & van den Heever, 2013; Seigel et al., 2013), and isolated convection (Fan et al., 2009; Han et al., 2012; Khain et al., 2004; Lebo & Seinfeld, 2011; Lee et al., 2008; Nissan & Toumi, 2013; Tao et al., 2007; van den Heever & Cotton, 2007). The current understanding of aerosol‐DCC interactions has been summarized in recent review papers (Altaratz et al., 2014; Fan et al., 2016; Rosenfeld et al., 2014; Tao et al., 2012).

### 1.2 Forest Fire Aerosols and Their Impacts on DCCs

Numerous studies have presented observational evidence for an increase in forest fire activity in the United States, such as fire frequency and active periods, in recent decades (e.g., Dennison et al., 2014; Jolly et al., 2015; Westerling et al., 2006), while others have projected future increase in fire activity (e.g., Flannigan et al., 2000; Liu et al., 2013). Dennison et al. (2014), for example, used satellite data to identify an increase in the frequency of large fires (burnt area larger than 1,000 acres) at an approximate rate of seven fires per year in the western United States for the period of 1984–2011. Liu et al. (2013) projected a future extension of the fire seasons in many locations in the United States, inferred from the projection of increased drought conditions. This projected increase in forest fire activity is a topic of increasing attention and active research, as the scientific community attempts to assess the full range of its possible impacts. In light of the aforementioned studies, understanding how smoke particles affect DCCs in the current climate, and in particular how these effects can be modeled, appears to be of crucial importance.

Particle emissions from forest fires have the following distinct characteristics: (1) Emissions are sporadic and episodic, yielding high emission rates that are confined to certain areas and seasons, and (2) emitted particles tend to have relatively low hygroscopicity  *κ* (Petters & Kreidenweis, 2007) due to large organic fractions (Kondo et al., 2011; Mallet et al., 2017; Moore et al., 2011; Reid et al., 2005) and also some contribution of black carbon (BC). The particles are often light absorbing, giving rise to the semi‐direct effect (Hansen et al., 1997). The exact characteristics, however, vary from case to case (Petters et al., 2009), and, while freshly emitted particles tend to exhibit a range of * κ* values, they often converge after atmospheric aging (Engelhart et al., 2012). Even though * κ* of fresh fire particles is relatively low, some observational studies suggested fire‐induced increases in CCN (Bougiatioti et al., 2016; Mallet et al., 2017; Wu et al., 2017). That is, large numbers of aerosol particles from forest fires were observed to contribute to cloud droplet formation.

Rosenfeld (1999) used TRMM satellite data to find a suppression of precipitation caused by biomass burning aerosols over Indonesia, as well as a resultant increase in cloud heights. They also hypothesized that the smoke particles may impact ice processes inside DCCs, which was partly confirmed by aircraft observations in the Amazon by Andreae et al. (2004). Studies on pyroclouds (Fromm et al., 2008; Lindsey & Fromm, 2008; Rosenfeld et al., 2007) found smaller ice crystal sizes in anvil clouds due to forest fires, which contributed to longer cloud lifetimes. In addition, certain previous studies also suggested that forest fire aerosols may alter lightning activity (Lyons et al., 1998; Murray et al., 2000). Altaratz et al. (2010) combined ground‐based lightning data and satellite observation to show evidence for a strong invigoration effect of forest fire aerosols on DCCs under low background aerosol concentrations and a strong semi‐direct effect suppressing convection under high background concentrations. These studies provide observational evidence for potentially strong impacts of smoke particles on DCCs' microphysical, dynamical, and radiative properties.

As for modeling studies, few studies have so far focused specifically on smoke‐DCC interactions on a cloud‐resolving scale; Luderer et al. (2006) used idealized simulations to test the impacts of heat, moisture, and CCN emissions from fires on a pyro‐cumulonimbus cloud, yet without explicitly including fire aerosols in their simulations. They concluded that the existence of fire aerosols delayed the freezing of droplets, which is consistent with Andreae et al. (2004), but in their simulations the delay actually led to slightly weaker convection due to decreased latent heat release. Grell et al. (2011) presented one of the earlier studies that used cloud‐resolving simulations with the Weather Research and Forecasting (WRF) model (Skamarock et al., 2008) coupled with Chemistry (WRF‐CHEM) (Grell et al., 2005) with forest fire input to investigate this topic. They found a substantial increase in the amount of convective precipitation and also in updraft velocities with the inclusion of local fire in their simulations over Alaska. Wu et al. (2011) used the same model to investigate the impacts of biomass burning aerosols on DCCs in South America and found a predominant importance of the aerosol radiative effect in reducing the diurnal cycle of precipitation. They explained their findings by daytime evaporation (“burn‐out”), which reduced precipitation, and subsequent condensation of the moisture at night, during which precipitation increased. Zhao et al. (2012) presented another study with the same model that investigated the impacts of non‐local aerosols on convection in the northeast United States and found low‐altitude (1.5–2.5 km) plumes to be most efficient in altering cloud properties (i.e., increasing the droplet number concentrations and lowering the precipitation by 30%). Their work was further extended in Zhao et al. (2014), using a global model (CAM5) to better constrain long‐range transport of fire aerosols in WRF‐CHEM simulations. In this study, they again found a decrease in the amount of precipitation by approximately 10% with the inclusion of fire input, likely due to the semi‐direct effect of the smoke plume that stabilized the atmosphere and reduced the convective instability. They also pointed out the importance of the smoke composition (i.e., ratio of BC to total aerosols). Saide et al. (2015) showed in their modeling study that the long‐range transport of biomass burning aerosols can increase the intensity of tornadoes in the United States mainly through their direct and semi‐direct effects, but this effect seems to depend on environmental conditions (Saide et al., 2016). Hodzic and Duvel (2018) separated microphysical and radiative effects of biomass burning aerosols in a case study of DCCs over the Borneo Island and found earlier suppression and later enhancement of precipitation from DCCs induced by the aerosol microphysical effects. These studies have made it clear that fire aerosols potentially have strong impacts on DCCs via their microphysical effects.

Whereas most studies have focused on changes in the number of aerosol particles due to forest fires, few have focused on the impact of fire emissions on aerosol hygroscopicity * κ*. An observational study by Bougiatioti et al. (2016) found a decrease in  *κ* when the atmosphere was influenced by fire events and also found that the importance of aerosol composition in determining droplet number concentrations depended on the distance from the fire source. Kawecki and Steiner (2018) recently reported changes in the spatial distribution of precipitation from a mesoscale convective system (MCS), when * κ* was modified in their numerical simulations. Fierce et al. (2017) showed that quantification of aerosol effects on clouds in large‐scale simulations may be affected by model representations of composition (that, in turn, affect * κ*). Therefore, the impacts of aerosol particles from forest fires on DCCs need to be assessed with respect to their effects on particle number concentrations (“the number effect” hereinafter), particle sizes (“the size effect”), and the overall * κ* (“the hygroscopicity effect”).

This study examined the microphysical effects of forest fire particles on DCCs while isolating the hygroscopicity effect in a semi‐idealized setting, using the cloud‐resolving WRF‐CHEM model. In addition, we investigated the impacts of a fire smoke plume that intersected DCCs at high altitude. These simulations were carried out with three different fire aerosol inputs in terms of emitted particle numbers, sizes, and/or mass, in order to examine the robustness of the findings. Note that the focus of this study is not on pyroclouds, but rather DCCs that formed in environments with abundant forest fire aerosols. Also, we did not investigate the sensitivity of the results to the background particle concentrations.

## 2 Data and Methods

The present study focuses on a particular case of DCCs observed downwind of forest fires in northeast Colorado. A series of cloud‐resolving simulations with WRF‐CHEM were performed, in order to investigate the sensitivities of the DCCs to changes in aerosol number and population‐averaged  *κ*. The case of interest and the observations made during the event are described in section 2.1, and the setups of the model simulations are described in section 2.2.

### 2.1 Case Description

**Figure 1:**
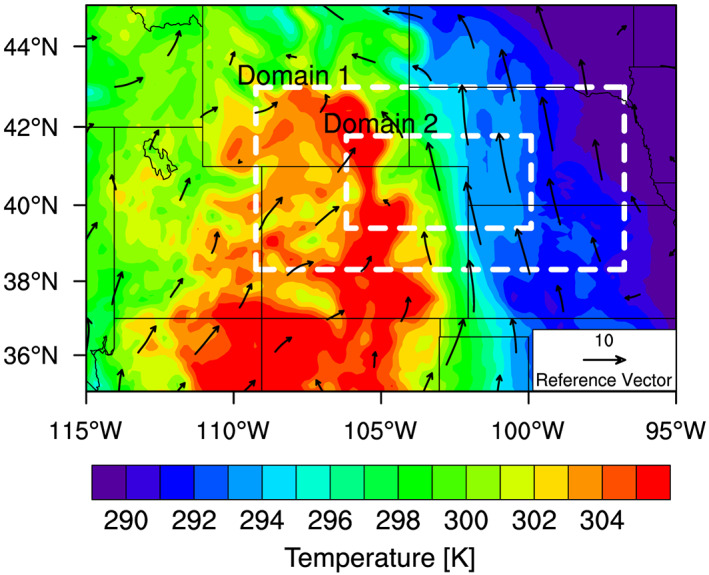
NAM 850 mb temperature (K) (contours) and winds (m s ^−1^) (vectors) at 1800 UTC on 22 June 2012, a few hours before the DCCs of interest developed. The approximate locations (i.e., minimum and maximum longitudes and latitudes) of the parent (“Domain 1”) and nested (“Domain 2”) domains for our WRF‐CHEM simulations are indicated by the white dashed rectangles.

**Figure 2:**
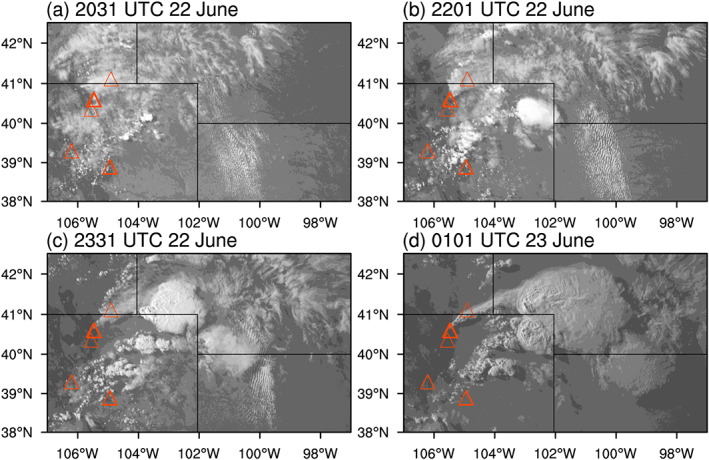
GOES satellite channel 1 visible images (UCAR/NCAR (Earth Observing Laboratory), 2012) centered around northeast Colorado at (a) 2031, (b) 2201, and (c) 2331 UTC on 22 June, and (d) 0101 UTC on 23 June. Orange triangles indicate the locations of forest fires between 1950 UTC on 22 June and 0250 UTC on 23 June, given by the FINN data. These GOES satellite data were obtained from the DC3 data website (https://data.eol.ucar.edu/master_list/?project=DC3).

**Figure 3:**
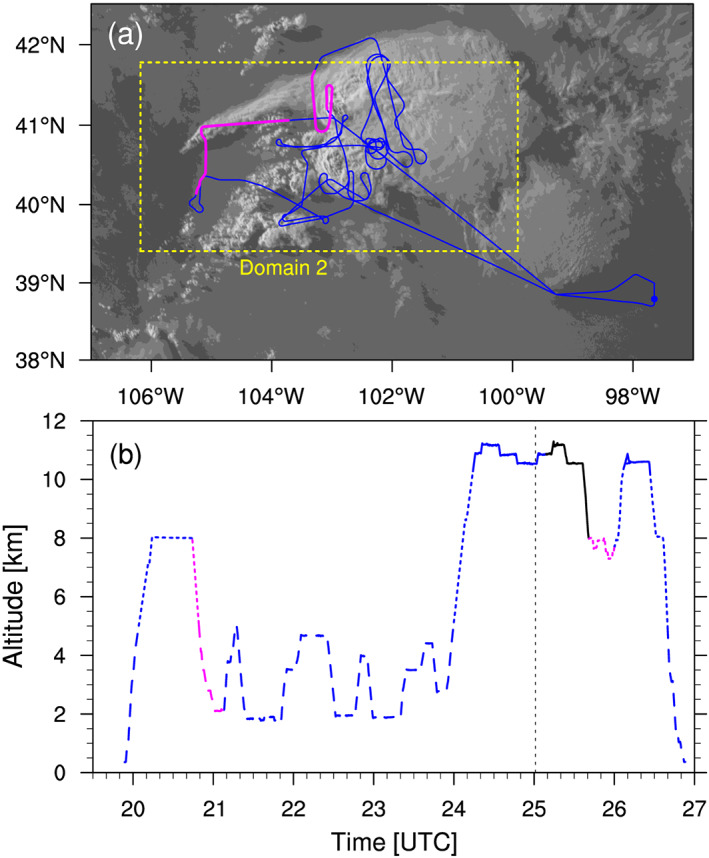
(a) Top‐down view of the DC8 flight path (blue) during the event (Chen et al., 2014), superimposed on the GOES satellite channel 1 visible image (UCAR/NCAR (Earth Observing Laboratory), 2012) at 0101 UTC on 23 June. The “in‐smoke” periods of the DC8 flight path (section 2.1) are colored in magenta. The yellow rectangle shows the location of the inner domain (Domain 2). (b) Time variation of the DC8 flight altitudes for the event (Chen et al., 2014), partly colored in magenta when DC8 was “in smoke”. The dashed, dotted, and solid lines indicate the warm (*T*  ≥ 0 °C), mixed‐phase ( −40 °C  < *T*  < 0 °C), and cold (*T*  ≤  −40 °C) temperature ranges, respectively. The black solid line shows the part where the temperature data is extremely low ( ∼200 K), likely an error. The black dashed line indicates the time when the GOES image in (a) was taken.

During the Deep Convective Clouds and Chemistry (DC3) field campaign (Barth et al., 2015) in 2012, DCCs developed in northeast Colorado, downwind of the High Park fire (Coen & Schroeder, 2015) on 22 June. The development of these DCCs were due in part to a warm low‐level southerly flow into the region (Figure 1). According to the DC3 mission summary for this event (available on the DC3 Field Catalog, see *Acknowledgements* for details), a few DCCs developed one after another in northeast Colorado starting at around 2030 UTC (Figure 2). During this event, the National Aeronautics and Space Administration (NASA) DC‐8 (DC8 hereinafter) and the National Science Foundation/National Center for Atmospheric Research (NSF/NCAR) Gulfstream V took in situ measurements for 7 hr from 2000 UTC on 22 June to 0300 UTC on 23 June. The DC8 aircraft also had flight legs that crossed the thick smoke plume from the High Park fire. Figure 3 shows the flight path, altitudes, and ambient temperatures of DC8 during this mission, according to the merged flight data (Chen et al., 2014) and satellite data (UCAR/NCAR (Earth Observing Laboratory), 2012). Figure 3b also shows that most of the measurements were made at around 3 and 11 km in altitude, which were the approximate heights of the convective inflow and outflow, respectively. The “in‐smoke” periods in this figure (about 2044–2108 and 2541‐2600 UTC) were somewhat arbitrarily defined, according to the mission summary (see *Acknowledgements*); DC8 flew inside the thick smoke plume during its descent to approximately 2 km, and hence, we defined the first in‐smoke period as the time from the beginning of the descent until the next ascent. It also flew through the thick smoke plume at around 7–8 km after a descent around 2545 UTC, and hence, we defined the second in‐smoke period as the time between the end of the large descent and 2600 UTC that was roughly when it started moving away from the DCCs (Figure 3a). The altitude of the smoke plume was approximately 7.5 km (i.e., mid‐troposphere) when it intersected the DCC. Although aerosols in the boundary layer are the main source of CCN at cloud base rather than those in the mid‐troposphere, the eastward transport of aerosols during and prior to this event must have increased the number of aerosols throughout the troposphere in the area of the DCC development. It should be pointed out that the DCCs started to develop in the late afternoon (approximately 2100 UTC, or 3 p.m. in Mountain Daylight Time) and kept developing until well after sunset (about 8:30 p.m.). As absorbing aerosols need sufficient time and strong sunlight to heat up the ambient air, we assumed a relatively small semi‐direct effect (Hansen et al., 1997) of the forest fire aerosols for this event. Indeed, there was no clear temperature rise inside or near the smoke plume, according to the DC8 temperature data (Figure S1 in the supporting information).

Observational data during this event was used to both validate and design our cloud‐resolving simulations. This includes the data from the following instruments; the Ultra High Sensitivity Aerosol Spectrometer (UHSAS) (Anderson, 2013; Cai et al., 2008) from NASA is able to count the number of aerosol particles sized between 0.060 and 1  μm by detecting the characteristics of laser light scattered by each aerosol particle inside the instrument. The CCN counter (Nenes, 2013; Roberts & Nenes, 2005) of the Georgia Institute of Technology optically counts the number of activated droplets after aerosols flow through a supersaturated column inside the instrument. The supersaturation varied within the range between 0.14% and 0.68% over time for this specific event. Aircraft data on thick liquid parts of the DCCs is scarce, likely due to the vigorous DCC updrafts that prevented aircraft from flying through the convective core. The UHSAS and the CCN counter data were split into the aforementioned in‐smoke periods and the rest, and compared to identify differences in aerosol properties between the plume of heavy smoke and the cleaner surrounding air. However, note that the latter also contained smoke particles, but not to the same extent as the plume itself. In addition to these in situ measurement data (including the aforementioned merged data (Chen et al., 2014)) and the satellite imagery (UCAR/NCAR (Earth Observing Laboratory), 2012), gridded precipitation data (Lin, 2011) was also utilized. All of these data sets were obtained from the DC3 website (https://data.eol.ucar.edu/master_list/?project=DC3).

### 2.2 WRF‐CHEM Simulations

We used the WRF‐CHEM model Version 3.6.1 (available for download: https://www2.mmm.ucar.edu/wrf/users/download/get_source.html) to assess the impacts of fire‐induced changes in aerosol number concentrations and population‐averaged  *κ* on the properties of the DCCs. The simulation domains (one parent and one nested) were designed to focus on northeast Colorado where the convection of interest was observed (section 2.1), as shown in Figure 1. In order to explicitly resolve DCCs, the horizontal grid spacing in the nested and parent domains was set to be 1 and 3 km, respectively, with 100 vertical levels in each domain. We turned off convective parameterizations in both domains, although it is still highly uncertain and controversial as to what horizontal resolution is high enough for explicitly resolving convection in simulations (e.g., Hong & Dudhia, 2012; Shin & Hong, 2013; Yu & Lee, 2010). The 6‐hourly North American Mesoscale Forecast System (NAM) data set (NCEP, 2012) provided initial and boundary conditions for our simulations at a 12‐km horizontal resolution. The simulations used the two‐moment Morrison microphysics scheme (Morrison et al., 2009), which includes five types of hydrometeors: cloud droplets, cloud ice, rain, snow, and graupel. The upper limit on ice crystal number concentrations in the original Morrison scheme was turned off. Without this modification, ice crystal number concentrations may not be as sensitive to changes in droplet number concentrations as they should be. We set the following physics options: the RRTMG longwave and shortwave radiation schemes (Iacono et al., 2008), the revised MM5 surface layer scheme (Jiménez et al., 2012), the unified NOAH land surface model (Tewari et al., 2004), and the University of Washington boundary layer scheme (Bretherton & Park, 2009).

Chemistry was simulated with the RADM2 gas‐phase chemical mechanism (Stockwell et al., 1990) and aerosol dynamics were simulated with the Modal Aerosol Dynamics Model for Europe (MADE) (Ackermann et al., 1998) that includes the Secondary Organic Aerosol Model (SORGAM) (Schell et al., 2001). The MADE/SORGAM scheme used in this study represents aerosol using a modal approach in which aerosol is separated into three modes (Aitken, accumulation, and coarse), assuming a lognormal size distribution with fixed width for each mode. All aerosols within each mode were assumed to contain identical mass fractions of constituent species, that is, each mode was modeled as a fully internal mixture. Therefore, when droplet activation was calculated based on Abdul‐Razzak and Ghan (2000) and thus the Köhler theory (Köhler, 1936), the same effective * κ* (Petters & Kreidenweis, 2007) determined by the volume ratios of aerosol species was used for all particles in the same mode. These assumptions (i.e., fully internal mixing and volume‐based * κ*) greatly simplify the calculations in the model, though in reality the aerosol population does not instantly mix uniformly. In order to isolate the microphysical effects of aerosol particles on clouds, aerosol effects through radiation were turned off in the inner domain. For simplicity and isolation of fire aerosol effects, photolysis and other emissions (both anthropogenic and biogenic) were turned off. The background aerosol concentrations were based initially on masses and geometric mean diameters (GMDs) (i.e., median diameters of lognormal *number* size distributions; 10 nm for Aitken, 70 nm for accumulation, and 1  μm for coarse) that were assumed for initial aerosol in the default configuration of MADE/SORGAM and subsequently evolved over time due to atmospheric processing (sulfate particles dominated later on). Wet scavenging was turned on in the simulations.

Impacts of aerosol particles from forest fires on DCCs, with respect to the number/size effect and the hygroscopicity effect, were investigated by running simulations with and without fire emissions while modifying * κ* at the same time. The assumption of uniform composition within each mode means that particles emitted into a particular mode instantly mix with all other particles in this mode, which may cause an artificial increase in the * κ* of freshly emitted particles (Fierce et al., 2017; Sánchez Gácita et al., 2017) or an artificial decrease in the  *κ* of non‐fire particles. Fire particles do mix with non‐fire particles in the atmosphere through coagulation, but it is unlikely that particles would become fully mixed by coagulation over such short timescales. This complexity can be better represented in models by having an additional fourth mode for hydrophobic accumulation‐mode particles, as some recent models do (e.g., Liu et al., 2016). The hygroscopicity effect that we quantified in this study is, therefore, applicable specifically to models that force fully internal mixing of fire and background particles within each mode. Under the fully external mixture approximation, wherein each particle contains only one species, the hygroscopicity effect defined in this study would reduce to zero, as different aerosol components would not mix and alter particles' effective  *κ*. However, emissions of particles with lower * κ* may still change the supersaturation state in the external‐mixing case (i.e., the competition effect, Ghan et al., 1998) even when the number/size effect is excluded, which eventually influences the number of activated droplets. This effect in the external‐mixing case was not estimated in this study, and we focus specifically on the hygroscopicity effect under the internal‐mixing assumption.

The simulations with forest fires included emissions according to the Fire Inventory from NCAR (FINN) (Wiedinmyer et al., 2011) data set (the version for the MOZART‐4 (Emmons et al., 2010)  chemical mechanism), which provides daily fire emission estimates over the United States at a high horizontal resolution of 1 km (available at https://bai.acom.ucar.edu/Data/fire/). The means by which the daily data was converted to hourly data is described in a report by Western Regional Air Partnership (see *Acknowledgements* for details). Using the 1‐D plume rise model (Freitas et al., 2007) embedded in WRF‐CHEM, vertical dispersion of fire‐related chemical species was calculated. Particles from the FINN were distributed across all three modes; for BC, organic carbon (OC), and unspeciated PM2.5 particles, they were distributed to the Aitken (25%) and the accumulation (75%) modes. The number emission rate of fire particles was calculated from the mass emission rate, assuming that, at the time of emission, each mode is represented by a lognormal size distribution with volume‐based GMD of 30 nm (Aitken), 300 nm (accumulation), and 6  μm (coarse) in the version of MADE/SORGAM that we used. The geometric standard deviations * σ*_*g*_ initially assumed for emitted particles were 1.70 (Aitken), 2.00 (accumulation), and 2.20 (coarse).

In the baseline simulations, with or without fire, the  *κ* for each mode was computed as the volume‐weighted * κ* of its constituent species (SO _4_, NO _3_, NH _4_: *κ*=0.5; elemental carbon : *κ*=10 ^−6^, dust : *κ*=0.1; sea salt: * κ*=1.16; OC and other anthropogenic : *κ*=0.14; all from MADE/SORGAM). However, the chemical composition of aerosol from biomass burning is highly variable and subject to large uncertainties (Kondo et al., 2011), which translates to large variability and uncertainty in the effective * κ* of biomass burning aerosol (e.g., Carrico et al., 2010). In order to explicitly investigate the hygroscopicity effect, * κ* was fixed in most of our simulations (i.e., the same * κ* for all particles across all three modes); * κ* was set to 0.25 for the case without fire (the NOFIRE case) and 0.10 for the cases with fire (the FIRE cases). The value of 0.25 was chosen to represent the range of population‐averaged * κ* during DC3 observed by Sorooshian et al. (2017) (e.g., see their Figure 2a). The lower value, 0.1, represents a lower bound on population‐averaged * κ*, representing a scenario in which the local aerosol population is strongly impacted by biomass burning emissions. Aerosol emitted from biomass burning contains large mass fractions of BC and OC and, therefore, tends to have a lower population‐averaged * κ* than the background * κ* of 0.25. Sorooshian et al. (2017) reported * κ* values  ∼0.10, except for the lowest vertical level, from a clear‐air sounding for the same case (see their Figure 3c). This low * κ*∼0.10 from the sounding, as compared to the approximate campaign‐mean * κ*∼0.25 (explained above) and the inflow/outflow‐mean * κ*∼0.25 for the same case (see their Table 2), is likely a result of the strong influence of fire particles during the same case. Even though * κ* values of biomass burning aerosols have large variability in space, time, and across fuels, * κ* values around 0.10 were observed in some experiments by Petters et al. (2009) (see their Figure 5). Engelhart et al. (2012) observed a wide range of * κ* for fire particles right after emission (0.065  < *κ* < 0.6), which converged to 0.2 ± 0.1 over time. Analysis of the time‐ and space‐invariable * κ* facilitated the attribution of the hygroscopicity effect separately from the number/size effect. This fixed‐ *κ* case also forced the absolute * κ* values to be closer to the observations by Sorooshian et al. (2017), in comparison with the  *κ* values diagnosed by WRF‐CHEM based on the simulated aerosol composition (see Figure S2). Because of the time‐invariant * κ*, the impact of temporal evolution of particle composition was not considered in our simulations.

**Figure 4:**
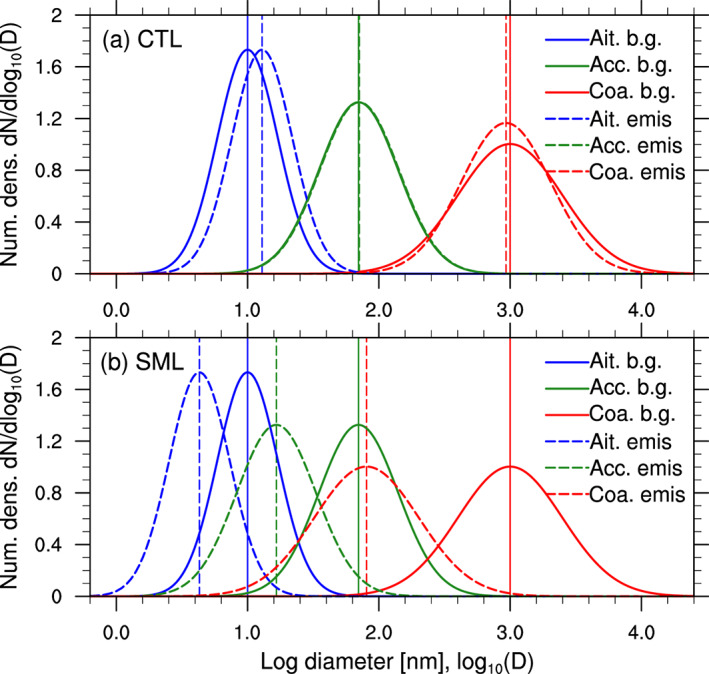
Number distributions of particle sizes in the (a) CTL and (b) SML runs, assumed for background (solid) and fire (dashed) particles in the Aitken (blue), accumulation (green), and coarse (red) modes at the time of initialization/emission. The vertical lines show the GMDs of the distributions in corresponding colors and patterns.

In addition to the FIRE and NOFIRE runs, we ran simulations including forest fires with * κ* set to 0.25 (the MODFIRE runs). These simulations represent fire‐induced changes in the aerosol number concentration and size distribution but assume that the fire particles are as hygroscopic as the background in order to isolate the impact of this number/size effect from the hygroscopicity effect. We defined the difference between NOFIRE and MODFIRE as the number/size effect (i.e., differences due to modified particle number concentrations and sizes) and the difference between FIRE and MODFIRE as the hygroscopicity effect (i.e., differences solely due to changes in * κ*). The combination of the two effects is the total fire effect and equivalent to the difference between NOFIRE and FIRE. Again, we emphasize that the hygroscopicity effect shown in this study is the result of the implicit assumption that particles mix instantly, which maximizes the influence of forest fire * κ* on other particles. We also note that the assumed *κ* of 0.10 for a fire‐influenced aerosol population is relatively low, though within the observed range of population‐average  *κ* (e.g., Sorooshian et al., 2017). The separation of the number effect and the size effect is challenging, as the numbers and sizes of particles change over time and space. Therefore, this study isolated the hygroscopicity effect and refers to the combined impact of changes in particle number concentrations and size distribution parameters as the number/size effect. We ran FIRE and MODFIRE runs with the default volume GMDs in one set (“CTL”). An additional set of sensitivity simulations (“SML”) was run in which the volume GMDs of emitted particles were reduced to 10 nm (Aitken), 70 nm (accumulation), and 1 μm (coarse). This additional set of simulations was used for testing the sensitivity of the simulated results to initial GMDs and number concentrations of fire particles that were assumed at the time of emission, while the total emitted mass was fixed. Figure 4 illustrates the differences in initial particle sizes assumed at the time of emission in the CTL and SML runs. For an additional sensitivity test, we ran another set of simulations in which fire aerosol mass from the FINN input was increased by a factor of 10 (“x10”). We used these three sets of simulations to evaluate the robustness of the simulated results. Table 1 lists the setting of the simulations.

**Table 1:** A List of the Simulations Conducted in This Study

					Volume GMDs at emission	
					(Aitken; accumulation;	
	Set	Name	*κ*	Fire input	coarse)	Droplet activation
1	Original	NOFIRE‐orig		Off	—	Default
2		FIRE‐CTL‐orig	Diagnosed	On	CTL (30 nm; 300 nm; 6 μm)	
3		FIRE‐SML‐orig	(Figure S2)		SML (10 nm; 70 nm; 1 μm)	
4		FIREx10‐orig			CTL (30 nm; 300 nm; 6 μm)	Enhanced∼7–8 km
5		NOFIRE	0.25	Off	—	Default
6	CTL	FIRE‐CTL	0.10	On	CTL (30 nm; 300 nm; 6 μm)	Default
7		MODFIRE‐CTL	0.25			
8		PLUME‐CTL	0.10			Enhanced∼7–8 km
9	SML	FIRE‐SML	0.10	On	SML (10 nm; 70 nm; 1 μm)	Default
10		MODFIRE‐SML	0.25			
11		PLUME‐SML	0.10			Enhanced∼7–8 km
12	x10	FIREx10	0.10	On (×10)	CTL (30 nm; 300 nm; 6 μm)	Default
13		MODFIREx10	0.25			
14		PLUMEx10	0.10			Enhanced∼7–8 km

In our simulations, the DCCs of interest developed slightly to the south of where strong convection was actually observed. As a result, the simulated DCC was influenced by fires located to the south of the High Park fire, as shown in section 3.2. In order to mimic the injection of the smoke plume into the DCCs at around 7–8 km (section 2.1), we also ran all the baseline FIRE simulations described previously (i.e., CTL, SML, and x10) while enhancing particle number concentrations by a factor of 20 exclusively for droplet activation between roughly 7 and 8 km (i.e., between 31st and 35th vertical levels) across the entire domain. More specifically, particle number concentrations that were used for droplet activation (i.e., *N*_*i*_ in equations (11) and (13) of  Abdul‐Razzak & Ghan, 2000) were multiplied by 20, while the particle concentrations used for the aerosol dynamics calculations remained unchanged. The value of 20 was based roughly on (1) an approximately 25‐fold increase in aerosol number concentrations inside the smoke plume between 7 and 8 km as measured by the UHSAS (Anderson, 2013) and also (2) an approximately thirteen‐fold increase in CCN number concentrations inside the smoke plume between 7 and 8 km as observed by the CCN counter (Nenes, 2013). These simulations with the idealized smoke plume are called PLUME‐CTL, PLUME‐SML, and PLUMEx10 (Table 1) and can be used for assessing the impacts of the enhanced droplet activation by the smoke plume inside the DCCs.

Because the DCCs of interest started to develop at around 2000 UTC on 22 June, our simulations started with 24 hr of spin‐up time from 1800 UTC on 21 June to 1800 UTC on 22 June, followed by 8 hr of analysis time from 1800 UTC on 22 June to 0200 UTC on 23 June. Even though the simulated convection was still producing surface rainfall at the end of the analysis time, it started partly moving out of the domain at around 0200 UTC, after which the comparison of the cloud properties between different runs was no longer meaningful. Therefore, all of the results in the following sections come from the nested domain during the 8 hr of the analysis time from 1800 UTC on 22 June to 0200 UTC on 23 June, except for accumulated surface precipitation pattern that we tracked in one simulation until 0600 UTC 23 June.

## 3 Results and Discussions

The following subsections compare simulations and observations (section 3.1) and present the results of the sensitivity tests with respect to cloud microphysics (section 3.2) and other cloud properties such as dynamics and radiation (section 3.3).

### 3.1 General Comparisons Between Simulations and Observations

**Figure 5:**
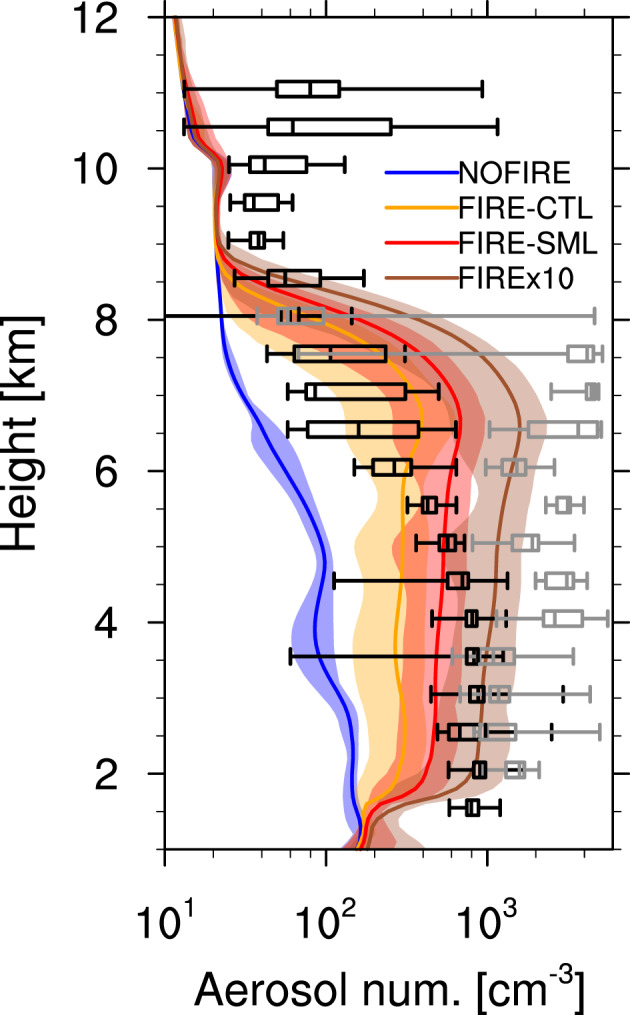
Vertical profiles of simulated interstitial particle number concentrations (cm ^−3^) in the NOFIRE (blue), FIRE‐CTL (orange), FIRE‐SML (red), and FIREx10 (brown) runs, averaged over the nested domain between 2000 UTC on 22 June and 0200 UTC on 23 June. The temporal minimum and maximum values at each height are indicated by the shading in each color. The profiles were vertically interpolated at every 100 m. The box plots show the UHSAS‐observed particle concentrations at ambient conditions (Anderson, 2013), binned every 500 m separately for in‐smoke (gray) and elsewhere (black) between 2000 UTC on 22 June and 0200 UTC on 23 June. Here the upper and lower ends of the whiskers are the maximum and minimum values, respectively. The particle size range is from 60 nm to 1  μm (diameter), equivalent to the UHSAS size range, for both the simulated profiles and the observed data. Simulation data that is (1) out of the observed longitude range (i.e., between 105.365°W and 106.180°W, which is not covered by the DC8 flight path; see Figure 3a), and/or (2) in grid boxes with non‐zero mass of precipitating hydrometeors (i.e., rain, snow, or graupel) was excluded.

**Figure 6:**
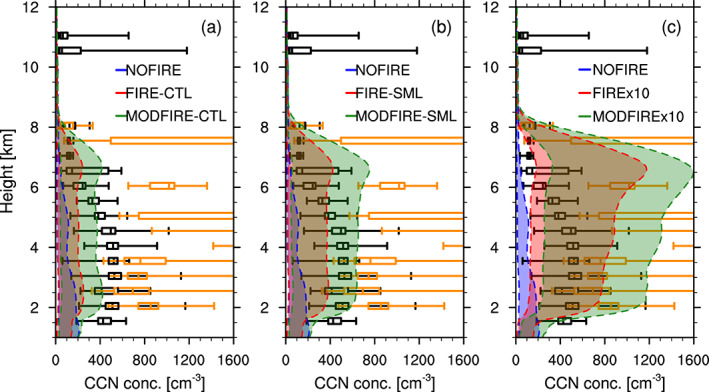
Vertical profiles of horizontal mean CCN concentrations (cm ^−3^) at 0.14% (lower edge of the shadings) and 0.68% (upper edge) supersaturations in (a) CTL, (b) SML, and (c) x10. These were estimated by firstly obtaining the critical diameters at supersaturation of 0.14% and 0.68%, respectively, using the equation (10) in Petters and Kreidenweis (2007) and the prescribed * κ* values (i.e., either 0.10 or 0.25). Second, the number of particles whose sizes exceed the critical diameter in each mode was calculated, given the model output of particle GMDs and the assumption of a lognormal distribution. Finally, the numbers of particles larger than the critical diameters in the three size modes were summed. Grid boxes with precipitating hydrometeors (i.e., rain, snow, and/or graupel) or out of the observed longitude range were excluded from the calculation. The averaging period is from 2000 UTC on 22 June to 0200 UTC on 23 June with a data interval of 10 min. The percentages of 0.14 and 0.68 were the minimum and maximum supersaturations, respectively, in the CCN counter (Nenes, 2013) during the event. The profiles were vertically interpolated at every 100 m. The box plots show the CCN concentrations (cm ^−3^) at ambient conditions observed by the CCN counter (Nenes, 2013) and binned every 500 m, separately for in‐smoke (orange) and elsewhere (black) between 2000 UTC on 22 June and 0200 UTC on 23 June. The upper and lower ends of the whiskers are the maximum and minimum values, respectively.

The comparison of observed and simulated aerosol number concentrations are shown in Figure 5; the profiles from the simulations are the averages over the inner simulation domain bounded by the minimum/maximum latitudes/longitudes of the DC8 flight path (excluding the precipitating grid boxes), including both in‐/near‐smoke regions and elsewhere. In the simulations with fire input, the particle concentrations are much higher than the NOFIRE runs up to about 8 km, which is consistent with the findings by Val Martin et al. (2013) (see their Figure 5). The observed particle concentrations in smoke show large variations, while those out of smoke show generally lower concentrations as expected. Although the simulated profiles mostly lie within the observed range (i.e., between in‐smoke and out‐of‐smoke observations), the concentrations in FIRE‐CTL and FIRE‐SML are lower than the observed concentrations below 6 and 5 km, respectively. Figure 6 compares CCN concentrations observed during DC3 on 22 June (Nenes, 2013) and estimated from the model output at 0.14% and 0.68% supersaturations (minimum and maximum during the observation, respectively). The simulated results are domain averages and the observations are in‐smoke/out‐of‐smoke averages during the flight. This figure indicates that the CCN number concentrations are reasonable in the x10 runs, as their estimated CCN concentrations fall mostly within the observed range between the in‐smoke (orange) maximum and out‐of‐smoke (black) minimum, regardless of * κ*. On the other hand, the other simulations (i.e., the NOFIRE simulation and the runs in CTL and SML) underestimate, on average, the CCN number concentrations compared to the observations, which is consistent with Figure 5. One can also see the impact of setting different initial aerosol sizes by comparing Figures 6a and 6b, as the SML runs show higher CCN concentrations than the CTL runs do, though the total mass of aerosol was unchanged. We consider the x10 runs as the most realistic case in terms of particle sizes and their number concentrations. There are several possible reasons that a tenfold increase in the emission rate was required to reproduce the observations; (1) the FINN data may have underestimated the total aerosol mass emitted from fires. (2) The FINN estimate for aerosol mass was reasonable but the assumed aerosol size at the time of emission in WRF‐CHEM was so large that it led to the underestimation of number concentrations. (3) The DCCs in our simulations developed to the south of where they were actually observed, and they may not have been as heavily influenced by the fires as they were in reality. (4) The wind pattern in our simulations did not transport as much particles as observed, both horizontally and vertically.

**Figure 7:**
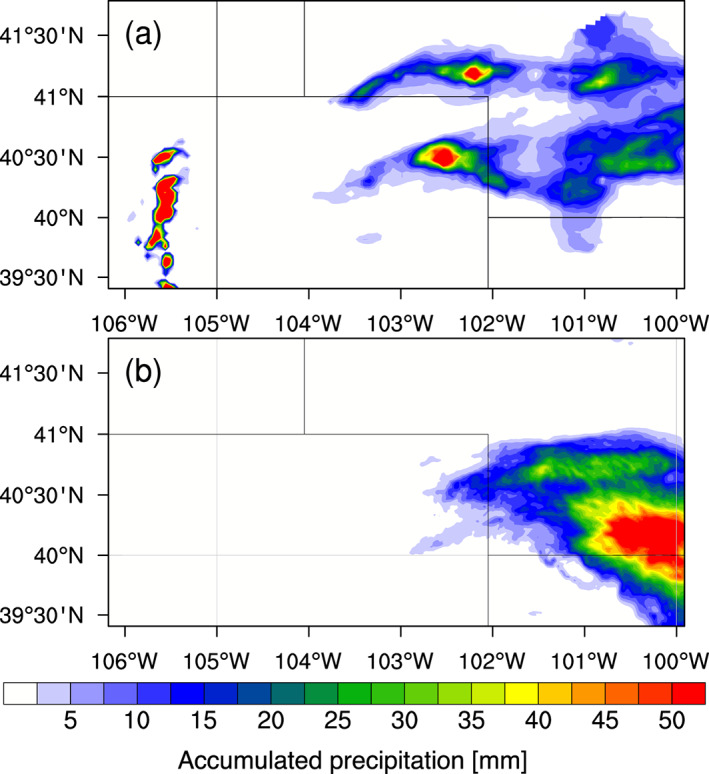
Distribution of accumulated surface precipitation [mm] between 1800 UTC on 22 June and 0600 UTC on 23 June, (a) observed by Lin (2011) and (b) simulated in the FIREx10 run. The observed high values between 105°W and 106°W are likely errors caused by forest fire particles.

**Figure 8:**
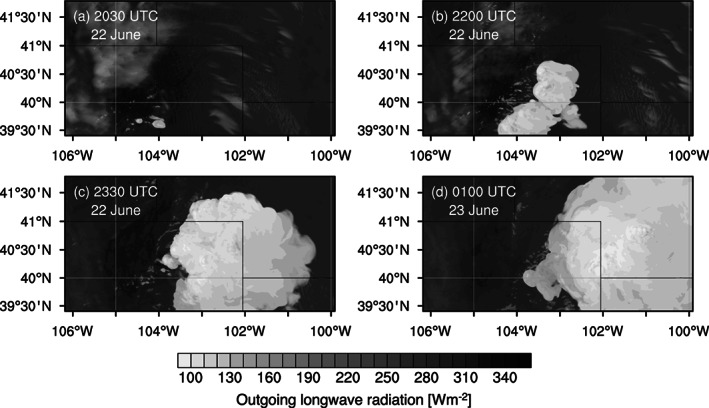
Simulated outgoing longwave radiation (OLR) (W m ^−2^) at (a) 2030, (b) 2200, and (c) 2330 UTC on 22 June, and (d) 0100 UTC on 23 June in the FIREx10 run. This can be qualitatively compared to Figure 2.

Figure 7a shows that the observed peak accumulated precipitation was approximately 50 mm in northeast Colorado. The observed (Figure 7a) and simulated (Figure 7b) distributions of surface precipitation show differences. While we acknowledge these differences between the simulated and observed clouds, the sensitivity tests on the simulated clouds are still meaningful and useful for investigating the number/size effect and the hygroscopicity effect, given the realistic timing (Figures 2 and 8) and intensity (Figure 7) of the DCCs. The target of this study is therefore DCCs developing in the general area of northeast Colorado under the influence of fire particles.

### 3.2 Fire Impacts on the Microphysical Characteristics of the DCCs

The microphysical characteristics of the simulated DCCs may change in response to the inclusion of forest fire particles, which can affect the aerosol loading and * κ* of the aerosol population. By comparing the different cloud‐resolving simulations listed in Table 1, this subsection discusses such fire‐induced changes in the DCC microphysical properties, which in turn may influence the dynamical and radiative features of the DCCs.

For our analyses, we defined convective cores as grid boxes with vertical velocities of 1.0 m s ^−1^ or higher, and water content (the sum of liquid drops and ice crystals) of 0.1 g m ^−3^ or higher at the same time. Convective anvils were defined as grid boxes with water content of 0.001 g m ^−3^ or higher at *T*  ≤  −40 °C, excluding convective cores. These definitions were set based on Hess et al. (1998) and Giangrande et al. (2016). See Figure S3 for the horizontal distributions of these grid boxes in the FIREx10 run and Figure S4 for the percentages of these grid boxes in the nested domain in all the runs. Note that an additional criterion (maximum ice crystal number concentrations in a column  ≥ 750 L ^−1^) for the convective anvil was imposed before 2330 UTC in order to exclude high ice clouds that did not originate from the DCCs (e.g., Figure 8a). The threshold value of 0.1 g m ^−3^ for water content in convective cores is relatively low, as compared to typical water contents in vigorous DCCs developing in moister environments such as in Rosenfeld and Lensky (1998), for example. The DCCs in our simulations developed in a much drier environment, and hence a very small fraction of the clouds has a very high (e.g.,  ≥ 1.0 g m ^−3^) liquid water content: see Figure S5 for the distributions of liquid water content. In order to capture the overall characteristics of relatively thick and convective parts of the clouds, the threshold of water content for convective cores was set to 0.1 g m ^−3^ in this study.

**Figure 9:**
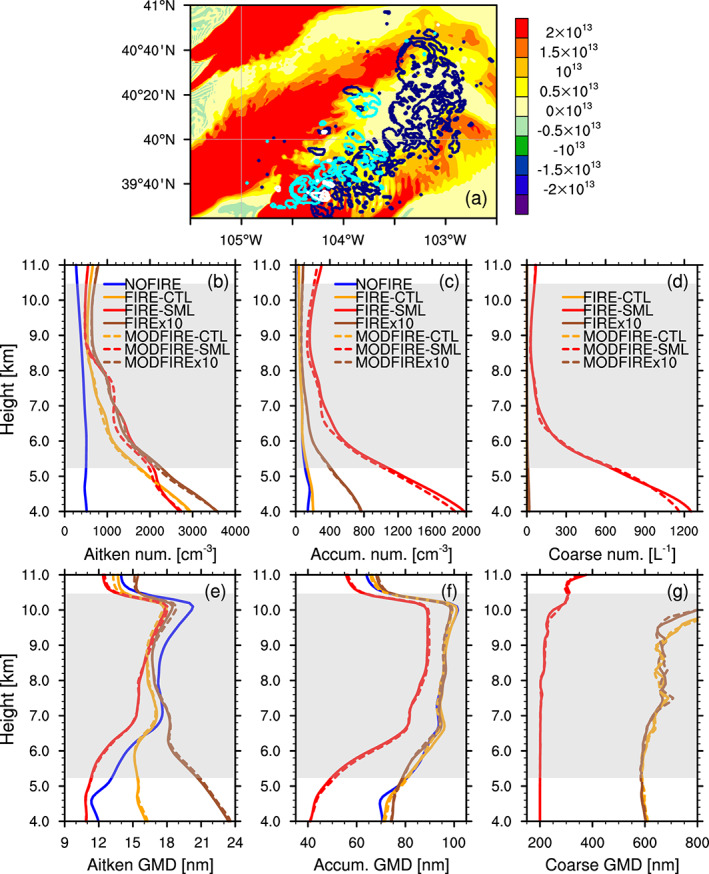
(a) Part of the simulation domain where the DCCs of interest developed, as the white, cyan, and dark blue contours indicate the existence of “convective core” grid boxes in the FIREx10 run at 2000, 2100, 2200 UTC, respectively. The background colors show the difference (FIREx10 −NOFIRE) in vertically integrated interstitial particle number concentrations (the sum of all three modes) (m ^−2^) at 2100 UTC on 22 June. (b–g) Vertical profiles of (b, e) Aitken (cm ^−3^), (c, f) accumulation (cm ^−3^), and (d, g) coarse mode (L ^−1^) interstitial aerosol number concentrations (b–d) and GMDs (e–g). These profiles are averages between 1950 UTC and 2150 UTC in the NOFIRE (blue), FIRE/MODFIRE‐CTL (orange), FIRE/MODFIRE‐SML (red), and FIRE/MODFIREx10 (brown) simulations. The calculation was done only in columns that included one or more “convective‐core” grid boxes in the subsequent output (10 min later). The coarse mode concentrations in the NOFIRE runs are too low to be shown in (d) and (g). The gray shading indicates the temporally and spatially averaged mixed‐phase temperature range inside convective cores in the NOFIRE run. The vertical profiles were vertically interpolated at every 100 m.

Simulated particle number concentrations are clearly different among different sets of simulations (i.e., CTL, SML, and x10; see Table 1), according to Figures 9b–9d. Note that the simulated aerosol concentrations and sizes in FIRE and MODFIRE are almost identical, as the solid and dashed lines in Figure 9 show, which confirms the negligible number/size differences between FIRE and MODFIRE. In addition to particle number concentrations, particle sizes are also different among different sets of simulations. For instance, when the FIREx10 and FIRE‐SML runs are compared, the former has many large Aitken‐mode particles (Figure 9e), whereas the latter has many small accumulation‐mode particles (Figure 9f). Both of these particle modes are large enough to be potentially important for droplet formation, as Figure 5 suggests.

**Figure 10:**
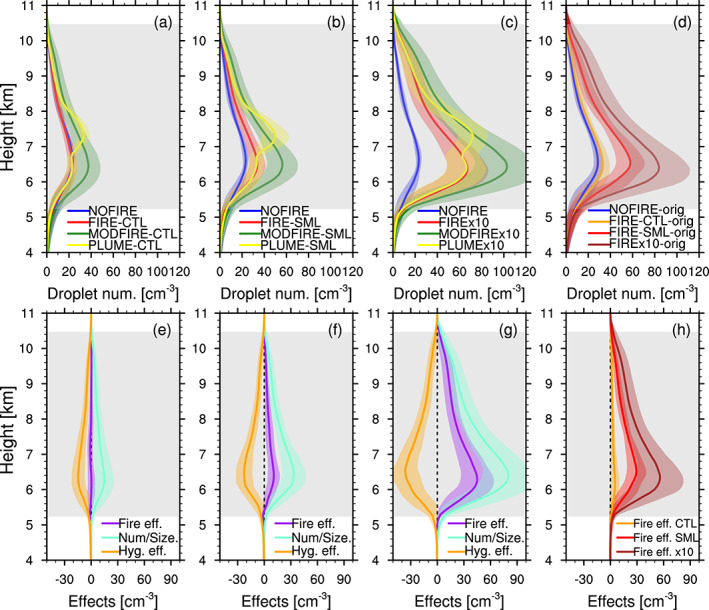
Vertical profiles of cloud droplet number concentrations (cm ^−3^) in the (a) CTL, (b) SML, (c) x10, and the (d) “‐orig” runs, averaged inside columns with one or more convective‐core grid boxes between 2000 and 2230 UTC (data every 10 min). The shading in each color shows  ± temporal standard deviation. The total fire effect (FIRE‐NOFIRE, purple), the number/size effect (MODFIRE‐NOFIRE, cyan), and the hygroscopicity effect (FIRE‐MODFIRE, orange) for the CTL, SML, and x10 are shown in (e)–(g), respectively. The total fire effects for the “‐orig” runs are shown in (h). The gray shading indicates the temporally and spatially averaged mixed‐phase temperature range inside convective cores in the NOFIRE run. These vertical profiles were vertically interpolated at every 100 m.

In terms of liquid droplet number concentrations, FIRE and MODFIRE show large differences (Figures 10a–10c and see Figure S6 for their time evolution); the MODFIRE runs show much higher droplet number concentrations, though liquid water content is similar across all the runs (Figure S7). The cloud‐base height, which lowers as the DCCs move eastward, is around 5 km on average (Figure S6). The number/size effect and the hygroscopicity effect on droplet number shown in Figures 10e–10g suggest that the hygroscopicity effect is so strong that it partially (SML and x10), or completely (CTL), offsets the number/size effect, on average. However, we stress here again that these simulations represent a particularly strong hygroscopicity effect. The variability in the number/size effect among the runs stems largely from differences in particle number concentrations (Figure 9). Therefore, if the * κ* values of the fire‐influenced aerosol population, as a whole, are, in fact, as low as 0.10, the hygroscopicity effect can be as strong as the number/size effect and suppress a fire‐induced droplet increase in simulations.

Although volume‐weighted bulk * κ* values in the “‐orig” runs (Figure S2) are higher than the observations by Sorooshian et al. (2017), droplet number concentrations in the “‐orig” runs (Figures 10d and 10h) show characteristics that are similar to the fixed‐ *κ* runs; (1) the fire effect (FIRE‐NOFIRE) is small, especially for the CTL runs, relative to the increase in aerosol number concentrations (Figures 9b–9d). (2) When the number/size effect is stronger such as in the SML runs, the overall droplet number concentrations increase as expected. Given the difficulty to isolate the hygroscopicity and number/size effects in the “‐orig” runs, the rest of the discussion focuses solely on the fixed‐ *κ* runs.

Note that the generally low droplet number concentrations are most likely the result of the combination of the small particle sizes and the low * κ*. According to our analyses, none of the droplet sink terms are large near cloud base (not shown). In our additional test simulation where the activated fraction of aerosol numbers was fixed (Aitken: 0.25, accumulation/coarse: 1.0) in the MODFIRE‐CTL configuration, droplet number concentrations are much higher than those in the original MODFIRE‐CTL simulation (cyan in Figure S8). From the result of this test simulation, it is inferred that the activated fraction is lowered in the original CTL runs (and likely in SML and x10 too) for some microphysical reason(s). We ran an additional test simulation in which * κ* was set to 1.16 (the * κ* assumed for sea salt in MADE/SORGAM), as well as another test run in which aerosol mode radii were forced to be much larger values (Aitken: 25 nm, accumulation: 250 nm, and coarse: 2.5  μm) but only in the calculation of aerosol activation. All of the three additional test simulations above were conducted in the MODFIRE‐CTL configurations (Table 1). We stress here that these additional test simulations were designed to isolate particular factors affecting simulated processes and are not intended to represent the true character of atmospheric aerosol. Clouds in the two additional simulations also show much higher droplet number concentrations in Figure S8 (purple and pink). These results suggest that in the CTL simulations the particle * κ* is too low and particles are too small (e.g., Figures 9e–9g) to be entirely activated even in the convective clouds. It should also be noted that the means by which the average profiles are calculated matter; the droplet number concentration profiles averaged among *columns* that include at least one convective‐core grid box, as in Figures 10 and S8a, show much lower concentrations than those averaged simply among convective‐core grid boxes as in Figure S8b. However, the former averaging is more suitable for qualitatively presenting the characteristics of the convective towers well.

**Figure 11:**
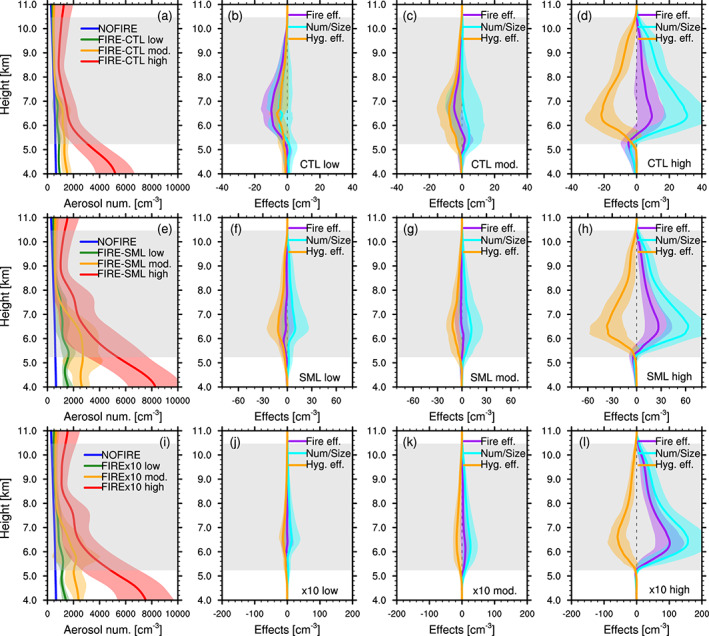
(a, e, and i) Vertical profiles of mean interstitial particle number concentrations (cm ^−3^) in NOFIRE (blue), FIRE “low” (green), FIRE “mod.” (orange), and FIRE “high” (red) in (a) CTL, (e) SML, and (i) x10, averaged between 1950 and 2220 UTC on 22 June. The definitions of “low", “mod.", and “high” columns are based on the mean interstitial particle number concentrations below 6 km; the averages in “low” columns are  < 1,060 cm ^−3^ (CTL),  < 1,850 cm ^−3^ (SML), and  < 1,575 cm ^−3^ (x10), in “high” columns they are  ≥ 1,850 cm ^−3^ (CTL),  ≥ 3,400 cm ^−3^ (SML), and  ≥ 2,900 cm ^−3^ (x10), and those between “low” and “high” in each set are categorized as “mod.” columns. (b–d, f–h, and j–l) The same plots as Figures 10e–10g but for (b, f, and j) “low", (c, g, and k) “mod.", and (d, h, and l) “high” categories in the (b–d) CTL, (f–h) SML, and (j–l) x10 simulations.  ± Temporal standard deviation is shown by the shadings in the corresponding colors. The gray shading indicates the temporally and spatially averaged mixed‐phase temperature range inside convective cores in the NOFIRE run. These vertical profiles were vertically interpolated at every 100 m.

The relative strengths of the number/size effect and the hygroscopicity effect were further investigated. As can be seen in Figure 9a, the DCCs developed under temporally and spatially varying aerosol loading, which is expected to affect the strength of the number/size effect. In order to investigate this dependence, model columns with one or more “convective‐core” grid boxes in FIRE/MODFIRE were split into three categories (“low", “mod.", and “high”), depending on the mean particle number concentrations below 6 km in the previous model output (i.e., 10 min before): The threshold concentrations (see the caption of Figure 11) were arbitrarily chosen so that the three categories have similar cumulative numbers of columns and therefore the average values among the columns are unlikely to be biased by the sample numbers. Figures 11a, 11e, and 11i show the average vertical profiles of aerosol concentrations in each category, along with the profile from the NOFIRE run as a reference. The total fire, number/size, and the hygrosocpicity effects were computed for each of the three categories (“low",“mod.", and “high”) and are shown in Figure 11 individually for the CTL (Figures 11a–11d), SML (Figures 11e–11h), and x10 runs (Figures 11i–11l). It is evident that the particle number concentrations affect the magnitudes of the number/size effect, and therefore the total fire effect, as well. Although the magnitude of the number/size effect generally increases with particle concentration, the concurrent increase in the hygroscopicity effect keeps the total fire effect no larger than  ∼63% of the number/size effect. The magnitudes of the hygroscopicity effect in relation to those of the number/size effect (100%) are summarized in Table 2. Although the magnitude varies, the relatively large contribution of the hygroscopicity effect found in Figure 10 is consistently seen in our simulations, regardless of the background particle concentrations.

**Table 2:** Summary of the Magnitude of the Hygroscopicity Effect in Relation to That of the Number/Size Effect (100%) in Each Case

		CTL	Low	SML	Low	x10	Low
			Mod.		Mod.		Mod.
			High		High		High
	Number/size effect	100%					
Total fire	(MODFIRE‐NOFIRE)						
effects							
(FIRE‐NOFIRE)	Hygroscopicity	−104%	(+137%)	−72%	−138%	−46%	−111%
	effect		−298%		−108%		−69%
	(FIRE‐MODFIRE)		−78%		−59%		−37%

*Note*. See the caption of Figure 11 for the definitions of “Low", “Mod.", and “High” in each set. These percentages (rounded to the nearest whole number) are based on the comparison of vertically (between 4 and 11 km) integrated mean liquid droplet number concentrations shown in Figures 10 and 11. CTL‐Low shows a positive value because its number/size effect is also slightly negative (see Figure 11b).

In Figures 10a and 10b, the impact of the idealized smoke plume at around 7–8 km can be seen by comparing the FIRE and PLUME runs. The twenty‐fold increase in aerosol number concentrations increased droplet number concentrations above 7 km, even though the increase amounts to less than a doubling of the droplet numbers in the FIRE runs, on average. Most of the droplet activation naturally takes place near cloud base where supersaturation maximizes, and hence additional particles at high altitudes only weakly affected droplet number concentrations (i.e., most of the cloud droplets must have been activated below and lifted upward). This finding suggests that the observed injection of the smoke plume into the DCCs likely had a small impact on droplets relative to its high particle number concentrations, although it might have had an influence on ice nucleation (e.g., Takeishi & Storelvmo, 2018). This effect of aerosols as INPs is beyond the scope of this study.

**Figure 12:**
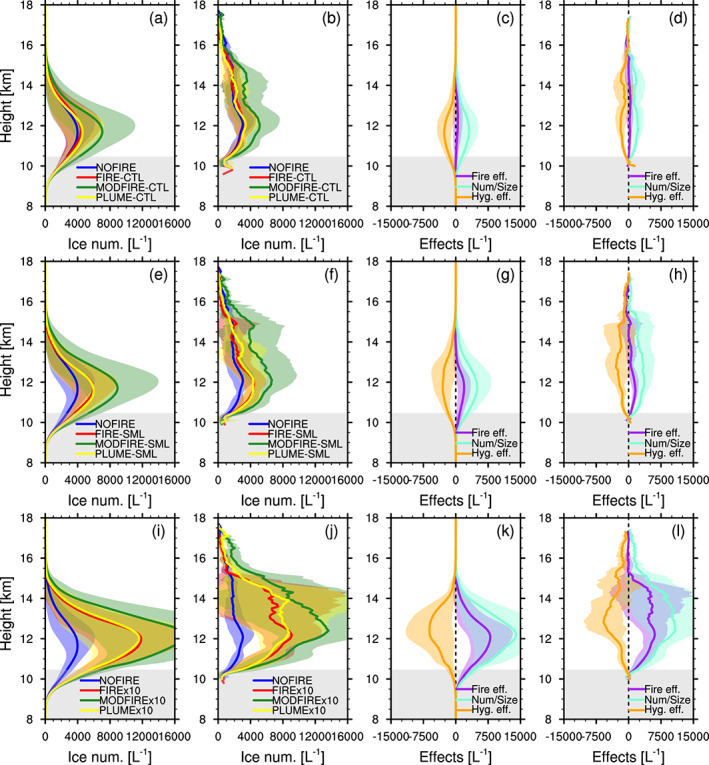
Vertical profiles of ice crystal number concentrations (L^-1^) inside (a, e, and i) columns with one or more convective‐core grid boxes and (b, f, and j) anvils in (a, b) CTL, (e, f) SML, and (i, j) x10, averaged between 2000 and 2230 UTC (data every 10 min). The total fire effect (FIRE‐NOFIRE, purple), the number/size effect (MODFIRE‐NOFIRE, cyan), and the hygroscopicity effect (FIRE‐MODFIRE, orange) are shown for (c, g, and k) convective cores columns and (d, h, and l) anvils in (c, d) CTL, (g, h) SML, and (k, l) x10. The shadings in corresponding colors show ± temporal standard deviation. The gray shading indicates the temporally and spatially averaged mixed‐phase temperature range inside convective cores in the NOFIRE run. These vertical profiles were vertically interpolated at every 100 m.

The differences in droplet number concentrations induced by the number/size effect and the hygroscopicity effect of aerosols also impact the cloud properties aloft. Figure 12 shows vertical profiles of ice crystal number concentrations, averaged inside convective‐core columns (a, e, and i) and anvil grid boxes (b, f, and j). The mean concentrations seem to vary with droplet number concentrations below (Figure 10). The number/size effect and the hygroscopicity effect of aerosols on ice also seem to be consistent with those for liquid droplets. The effect of the idealized plume (i.e., the difference between FIRE and PLUME) is very small for ice number concentrations, as can be expected from the relatively small difference in droplet number concentrations (Figure 10).

**Figure 13:**
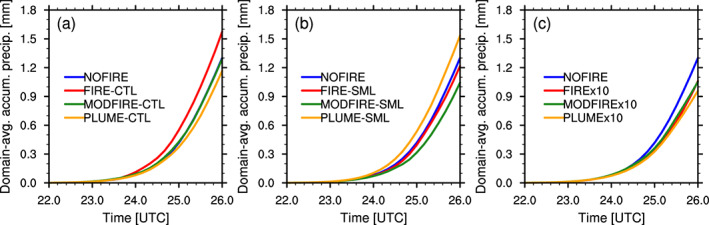
Time series of the simulated domain‐averaged accumulated surface precipitation (mm) over the nested domain since 1800 UTC on 22 June in the (a) CTL, (b) SML, and (c) x10 simulations.

**Figure 14:**
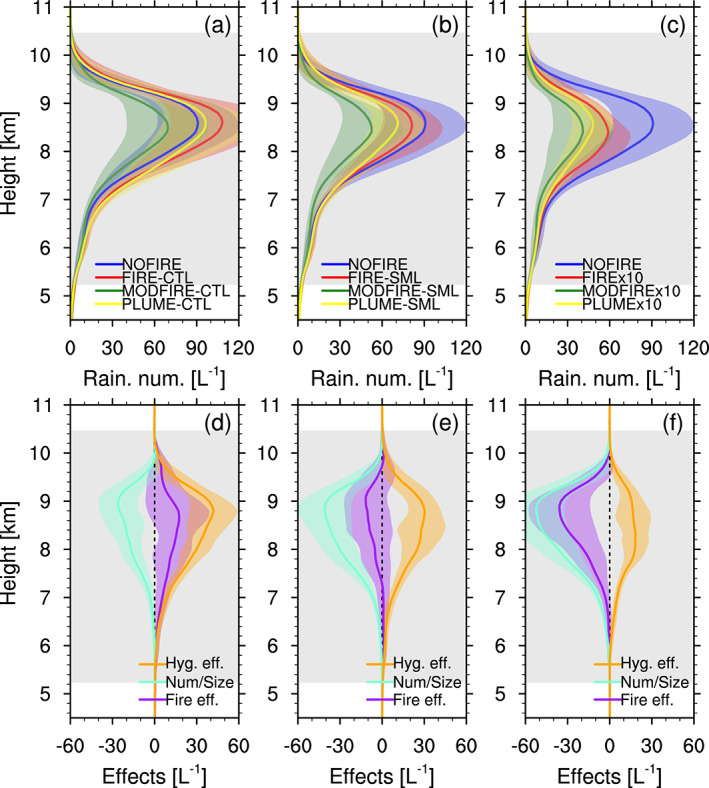
Vertical profiles of raindrop number concentrations (L^−1^) in (a) CTL, (b) SML, (c) x10, averaged inside columns with one or more convective‐core grid boxes between 2000 and 2230 UTC (data every 10 min). The total fire effect (FIRE‐NOFIRE, purple), the number/size effect (MODFIRE‐NOFIRE, cyan), and the hygroscopicity effect (FIRE‐MODFIRE, orange) are shown in (d)‐(f) for the CTL, SML, and x10 simulations, respectively. The shadings in corresponding colors show  ± temporal standard deviation. The gray shading indicates the temporally and spatially averaged mixed‐phase temperature range inside convective cores in the NOFIRE run. These vertical profiles were vertically interpolated at every 100 m.

Precipitation from the simulated DCCs occurs mostly through snow and graupel, which melt before reaching the surface (see Figure S9). Although the aerosol effects are apparent on ice crystal *number* concentrations, their impacts on ice crystal *mass* is weak (Figure S10). Changes in the amount of precipitation, as compared to NOFIRE, are not consistent among CTL, SML, and x10 (Figure 13). Number concentrations of raindrops change inversely with liquid droplet number concentrations (Figure 14). This can be explained by the increased/decreased droplets‐to‐rain autoconversion rates due to larger/smaller droplet sizes. This may lead to warm‐rain suppression, but the overall precipitation is dominated by graupel and snow that melt before reaching the surface (Figure S9). The insensitivity of snow and graupel mass to aerosol perturbation may be caused by the saturation adjustment, the strong upward motion that makes most/all liquid mass freeze regardless of droplet number concentrations, and/or multiple opposing processes that “compensate” for the loss/gain of snow and graupel mass. It is therefore clear that the aerosol effects on liquid droplets and raindrops cannot be simply translated into changes in the amounts of surface precipitation for the DCCs of interest in this study, mainly because of the dominance of ice processes.

### 3.3 Fire Impacts on the Dynamical and Radiative Characteristics of the DCCs

Invigoration of convection with increased aerosol particles, as introduced in section 1.1, is not applicable to the DCCs in this study, as the warm part of the cloud (*T*  ≥ 0 °C) is not sufficiently deep (e.g., Altaratz et al., 2014). The vertical profiles of updraft speed are therefore quite similar among different runs and do not show the invigoration effect (Figure S11). Due partly to this similarity in convective vigor, horizontally spreading anvil clouds also have similar areal coverage among the runs (Figure S12). It should be noted here that, however, some studies such as Fan et al. (2013) have shown that the microphysical effects of aerosol (i.e., altered hydrometeor sizes) can have a stronger overall impact on the convective anvil properties than the thermodynamic effects of aerosols (i.e., invigoration) do. Indeed, although the simulated DCCs have very similar dynamical characteristics (e.g., Figure S11), their anvils show somewhat different radiative characteristics among different runs as explained below.

**Figure 15:**
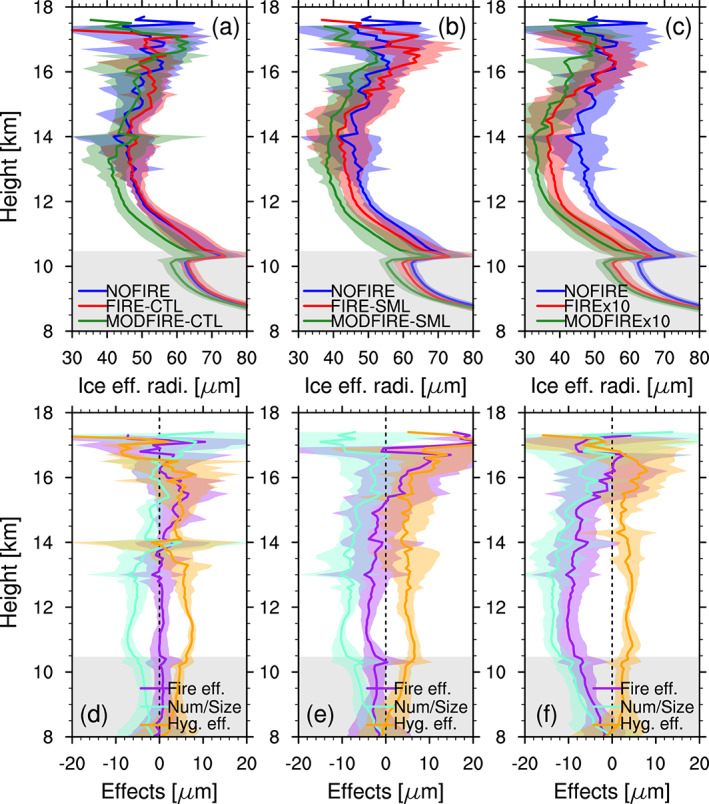
Vertical profiles of ice crystal effective radius (μm) in (a) CTL, (b) SML, and (c) x10, averaged inside grid boxes of either convective cores or anvils between 2000 and 2230 UTC (data every 10 min). The total fire effect (FIRE‐NOFIRE, purple), the number/size effect (MODFIRE‐NOFIRE, cyan), and the hygroscopicity effect (FIRE‐MODFIRE, orange) are shown in (d)–(f) for the CTL, SML, and x10 simulations, respectively. The shadings in corresponding colors show  ± temporal standard deviation. The gray shading indicates the temporally and spatially averaged mixed‐phase temperature range inside convective cores in the NOFIRE run. These vertical profiles were vertically interpolated at every 100 m.

**Figure 16:**
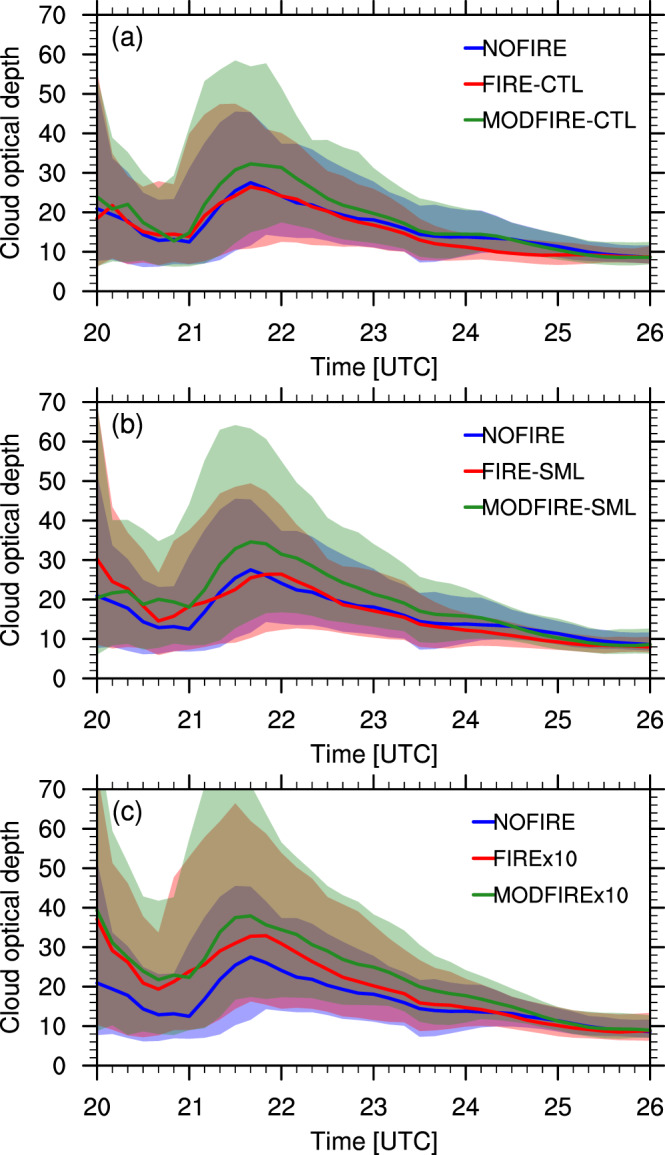
Time series of median cloud optical depth over the nested domain in the NOFIRE (blue), FIRE (red), and MODFIRE (green) runs in (a) CTL, (b) SML, (c) x10. The lower‐quartile and the upper‐quartile of the horizontal distribution at each time are indicated by the shadings in the corresponding colors. Note that the values were estimated from the model output of cloud droplet and cloud ice mass and sizes, and were not output by the model. The computation was done only in grid boxes with either convective cores or anvils.

In section 3.2, we showed that the increased particle concentrations led to increased droplet and ice crystal number concentrations, though their mass concentrations remained more or less unchanged (Figures S7 and S10). This means that the effective radius of ice crystals is smaller for the runs with higher ice number concentrations, as Figure 15 shows. As expected for clouds with similar ice water contents, runs with smaller ice crystal effective radii show larger cloud optical depths (Figures 16 and S13). This is clear only during the early stage of ice cloud formation when there are large differences in ice crystal number concentrations among the runs (Figure S14). Thus, convective clouds with higher droplet number concentrations below produced optically thicker anvil clouds in our simulations, as apparent in the MODFIRE runs (Figure S13). Although in our simulations the differences are limited to the first few hours (Figure 16) and contribution of precipitating hydrometeors to optical depth is not taken into consideration, this may have non‐negligible impacts on climate in the long run, when averaged over many DCCs.

Thus, increased aerosol particles originating from fires may impact microphysical and radiative properties of DCCs, and the impacts are crucially dependent on the number, size, and * κ* of the aerosol particles.

## 4 Conclusions

The CCN effects of forest fire aerosol on DCCs were investigated using the WRF‐CHEM model. This study emphasizes the importance of accurately representing * κ* to understand and assess the impacts of fire‐induced aerosol particles on DCCs. We also showed that models that assume fully internal mixing of aerosol may overestimate the hygroscopicity effect when the aerosol population is heavily influenced by fires, which may lead to underestimation of liquid droplet number concentrations in models. Important findings from this study can be summarized as follows:

1. When the effects of forest fire aerosols were divided into the number/size effect (i.e., an increase in the total number of aerosols and/or a change in aerosol sizes) and the hygroscopicity effect (i.e., a decrease in the population‐averaged particle * κ*), their relative strengths varied from case to case but the magnitude of the hygroscopicity effect was  ∼37% or higher (opposite sign) when compared to the magnitude of the number/size effect (100%). Such a strong hygroscopicity effect would be particular for cases in which fire and background particles are in the accumulation-mode size range, the size range for which particles are most sensitive to changes in  *κ*. As observations often suggest that number/size is more important than composition in determining CCN activity (e.g., Andreae et al., 2004), this relatively large contribution by the hygroscopicity effect is likely stronger than what would be observed in nature. The strong impact of hygroscopicity is likely the result of the common model approximation that particles are fully mixed within each mode. This exaggeration of the hygroscopicity effect in models can be avoided when models distinguish fresh and less hygroscopic particles from aged and more hygroscopic aerosol.
2. An increase in aerosol particles led to increased droplet and ice crystal number concentrations in the DCCs, especially for emissions with relatively high  *κ*. Number concentrations of raindrops were reduced by an increase in droplets, but this had negligible impacts on the mass concentrations of rain and the amount of surface precipitation. These results are relevant for cases where fire particles have higher  *κ* values due to aging processes, for example.
3. Increased ice crystal number concentrations in the DCCs led to increased anvil cloud optical thickness during the initial stage of the DCC development.
4. The PLUME runs suggested that the injection of the smoke plume into the DCCs at high altitudes (i.e., between 7 and 8 km) likely had a small impact on cloud droplet number concentrations relative to its aerosol particle numbers, as the majority of droplet activation would have taken place well below the injection heights.
5. The weak sensitivity of surface precipitation to the aerosol perturbation may be due to the relatively unchanged mass concentrations of snow and graupel.

On the other hand, the following points were not addressed in this study:

1. The semi‐direct effect of forest fire aerosols, which we assumed to be small in this study due to the late afternoon‐evening convection, may in some cases play a major role in changing the vertical temperature profile and hence cloud properties.
2. Although it was shown that fire particles may affect the microphysical and radiative properties of DCCs, discussing the general applicability of this aerosol effect requires sampling and statistics of many more convective clouds in the same area.

The findings from this study suggest that accurate representation of * κ* in numerical weather prediction and climate models may be essential for accurate representation of DCCs. Furthermore, given the potentially large impact of fire aerosols on DCC radiative properties, it is important to represent aerosol‐DCC interactions in global climate models (GCMs). This is currently missing in most state‐of‐the‐art GCMs. Finally, given the importance of the size and * κ* of fire particles reported here, future field observations that provide additional information on these quantities would be extremely valuable.

## Supporting information



Supporting Information S1

Data Set S1
