# Supplementary material for: Disentangling the Microphysical Effects of Fire Particles on Convective Clouds Through A Case Study
Source: J Geophys Res Atmos. 2020 Jun 16;125(12):e2019JD031890. doi: 10.1029/2019JD031890 (PMC7379315; doi:10.1029/2019JD031890)
Supplement: Supplementary file 1 — Supporting Information S1 [file JGRD-125-e2019JD031890-s001.pdf]

Supporting Information for "Disentangling the microphysical effects of fire particles on convective clouds through a case study"

Azusa Takeishi[1,2], Trude Storelvmo[3], and Laura Fierce[4]

Contents of this file

Corresponding author: Azusa Takeishi, Laboratoire d'Aérologie, University of Toulouse/CNRS, Toulouse, France (azusa.takeishi@aero.obs-mip.fr)

[1]Department of Geology and Geophysics, Yale University, New Haven, Connecticut, U.S.A.

[2]Currently at Laboratoire d'Aérologie, University of Toulouse/CNRS, Toulouse, France

[3]Department of Geosciences, University of Oslo, Oslo, Norway

[4]Environmental and Climate Sciences Department, Brookhaven National Laboratory, Upton, New York, U.S.A.

Figures S1 to S14

Additional Supporting Information (Files uploaded separately)

Caption for Dataset S1

Introduction

This supporting information provides Figures S1-S14 that are relevant to the main text but not central to the discussions, as well as the caption for Dataset S1 that contains namelist and module files for the presented WRF-CHEM simulations (uploaded separately).

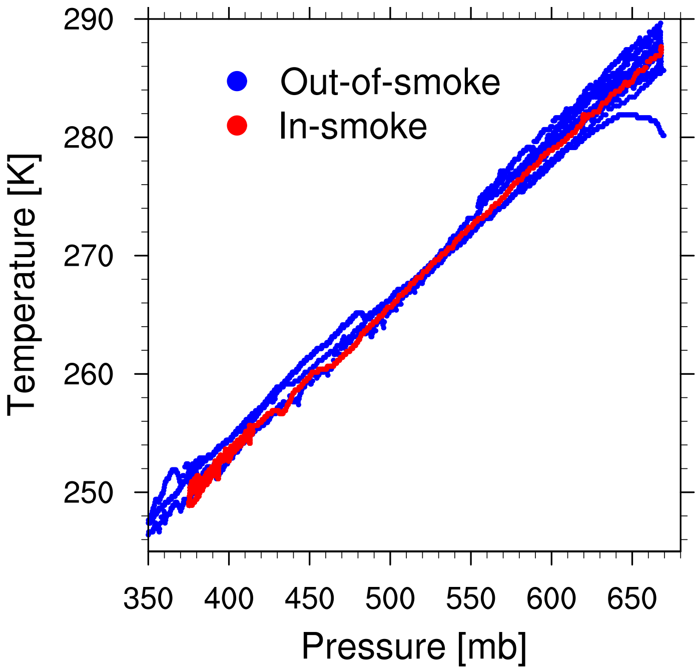

Figure S1. DC8 ambient temperature and pressure measured during the mission on 22 June 2012 (blue), while in-smoke data is colored in red. For the purpose of comparing in-smoke and out-of-smoke data, this figure specifically focuses on the temperature and pressure range in which smoke was detected (350-670 mb and 245-290 K), and also the data collected above 3.5 km in altitude; below this height, a sharp horizontal temperature gradient due to the warm southerly flow impedes the fair comparison between in- and out-of-smoke, as slightly indicated by the departure of the temperature in one flight leg near 670 mb. There is no apparent warming observed in smoke, suggesting a small semi-direct effect due to the smoke.

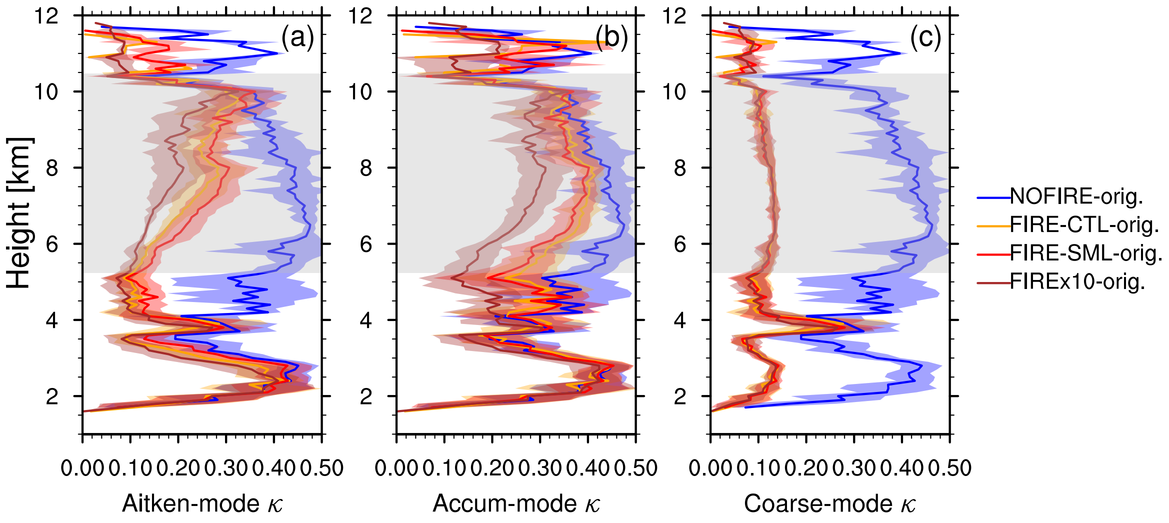

Figure S2. Vertical profiles of hygroscopicity κ in the NOFIRE-orig (blue), FIRE-CTL-orig (orange), FIRE-SML-orig (red), and FIREx10-orig (brown) runs for (a) Aitken-, (b) accumulation-, and (c) coarse-mode particles. The profiles are the averages in grid boxes where aerosols get activated inside columns that have one or more "convective-core" grid boxes in the subsequent model output (i.e., 10 minutes later) between 1950 UTC and 2220 UTC on 22 June. $\pm$ Temporal standard deviation for each profile is shown by the shading in the corresponding color. The grey shading indicates the temporally and spatially averaged mixed-phase temperature range averaged inside convective cores in the NOFIRE run. These profiles were vertically interpolated at every 100 m.

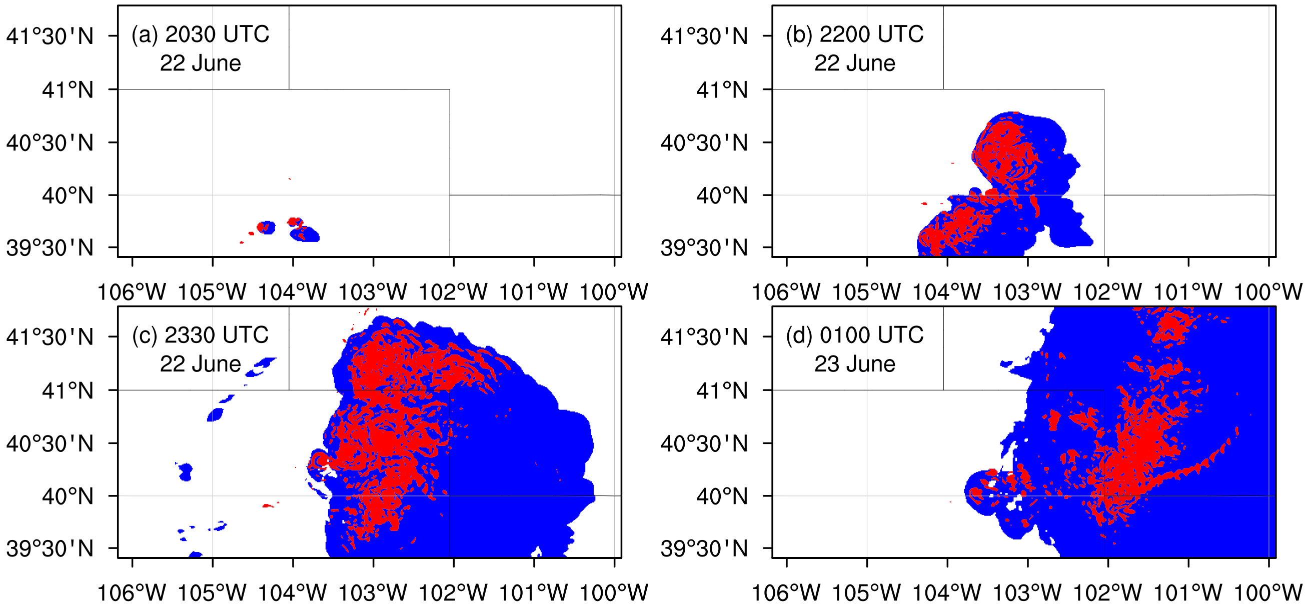

Figure S3. Snap shots of the top-down view of grid boxes categorized as convective cores (red) and anvil clouds (blue) at (a) 2030 UTC, (b) 2200 UTC, (c) and 2330 UTC on 22 June and (d) 0100 UTC on 23 June in the FIREx10 run. Note that columns with both cores and anvils are colored in red here.

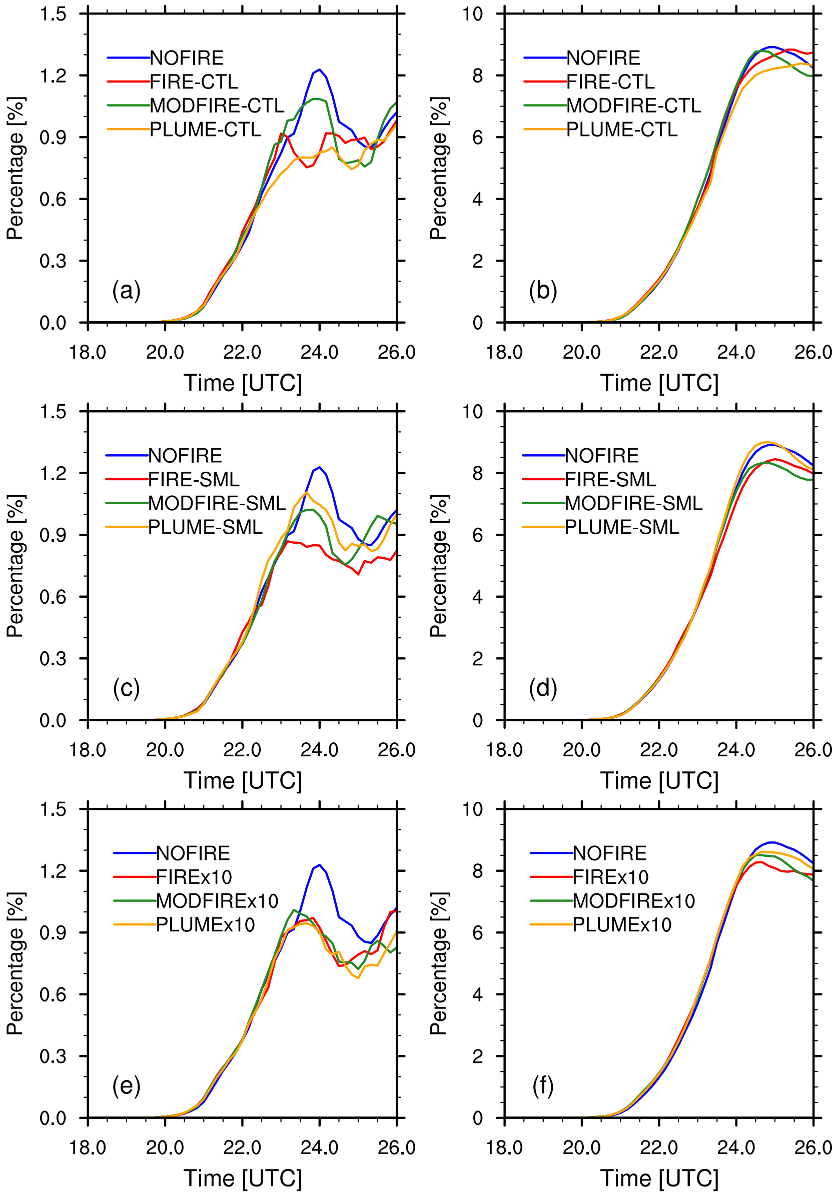

Figure S4. Time series of the percentages [%] of grid boxes categorized as (a,c,e) convective cores and (b,d,f) convective anvils in the nested domain in the (a,b) CTL, (c,d) SML, and (e,f) x10 runs.

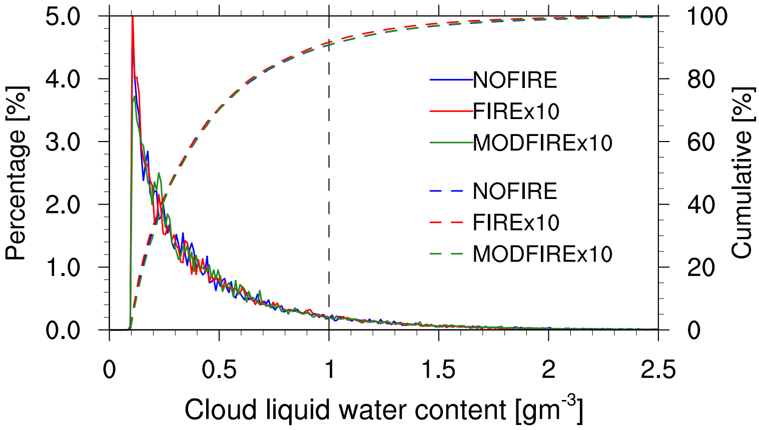

Figure S5. Temporally averaged distributions [%] of simulated cloud *liquid* water content (solid, right y-axis) in x10 and their cumulative percentages [%] (dashed, left y-axis), binned every 0.01 gm^{-3}. The data was obtained from "convective-core" grid boxes between 5.5 km and 6.5 km in altitude where droplet number concentrations maximize in most of the runs, and the distributions shown are the averages between 20 UTC and 2230 UTC. The vertical black dashed line indicates 1.0 gm^{-3} as a reference threshold of high liquid water content. The CTL and SML simulations show very similar results, and therefore are not shown.

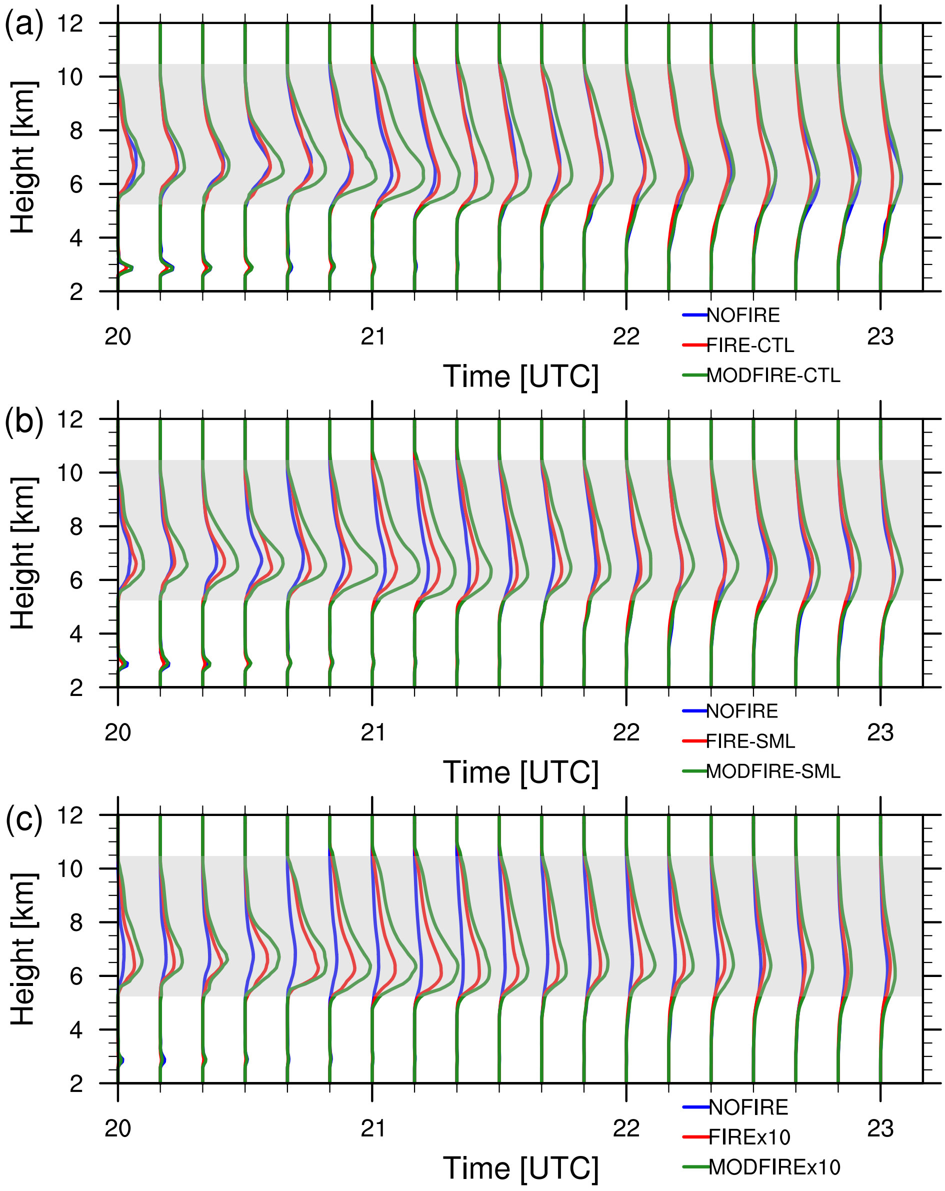

Figure S6. Changes in the vertical profiles of droplet number concentrations (averaged inside columns with one or more convective-core grid boxes) over time, from 20 UTC to 23 UTC on 22 June in the (a) CTL, (b) SML, and (c) x10 runs. Since this figure focuses on the time series of vertical profiles and not the actual concentrations, different sets of simulations (e.g., CTL and SML) were normalized by different values. The grey shading indicates the temporally and spatially averaged mixed-phase temperature range inside convective cores in the NOFIRE run. These profiles were vertically interpolated at every 100 m.

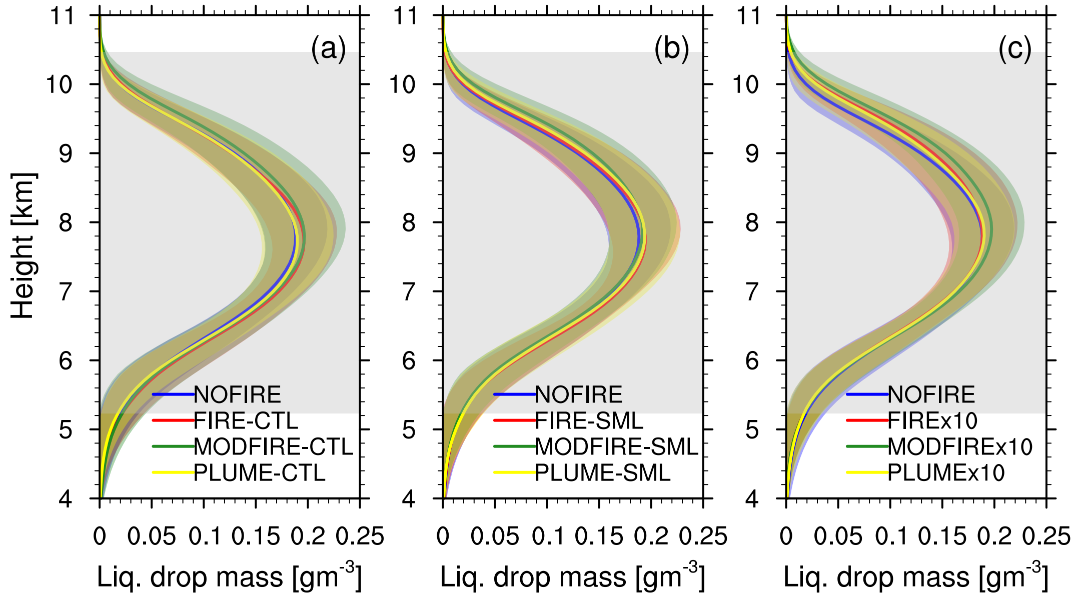

Figure S7. Vertical profiles of liquid cloud mass concentrations [gm^{-3}] in the (a) CTL, (b) SML, and (c) x10 runs averaged among columns with one or more convective-core grid boxes between 20 UTC and 2230 UTC on 22 June (data every 10 minutes). The shadings show ± temporal standard deviation. The grey shading indicates the temporally and spatially averaged mixed-phase temperature range inside convective cores in the NOFIRE run. These vertical profiles were vertically interpolated at every 100 m.

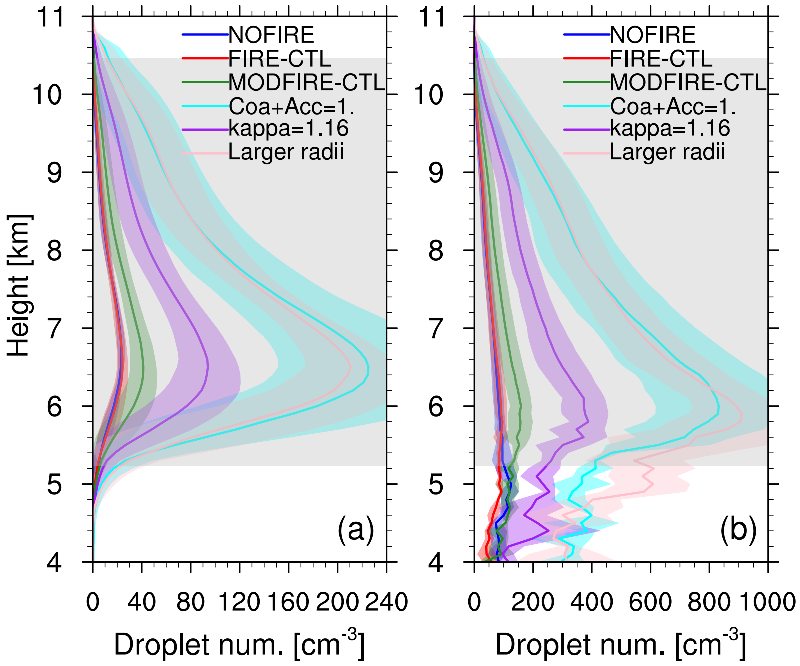

Figure S8. Vertical profiles of cloud droplet number concentrations [cm^{-3}] in NOFIRE (blue), FIRE-CTL (red), and MODFIRE-CTL (green), as in Figure 10a. Additionally, the profile from a test simulation in which the fractions of activation for aerosol numbers were set to 1 (accumulation and coarse) and 0.25 (Aitken) is shown in cyan, a simulation in which κ of all particles was set to 1.16 is shown in purple, and a simulation in which the mode radii used for the calculation of droplet activation were set to 25 nm (Aitken), 250 nm (accumulation), and 2.5 μm (coarse) is shown in pink. These three additional simulations were all conducted in the MODFIRE-CTL configurations (Table 1). The profiles are averages (a) inside columns with one or more convective-core grid boxes and (b) only among convective-core grid boxes, from 20 to 2230 UTC (data every 10 minutes). The shading in each color shows $\pm$ temporal standard deviation. The grey shading indicates the temporally and spatially averaged mixed-phase temperature range inside convective cores in the NOFIRE run. These vertical profiles were vertically interpolated at every 100 m.

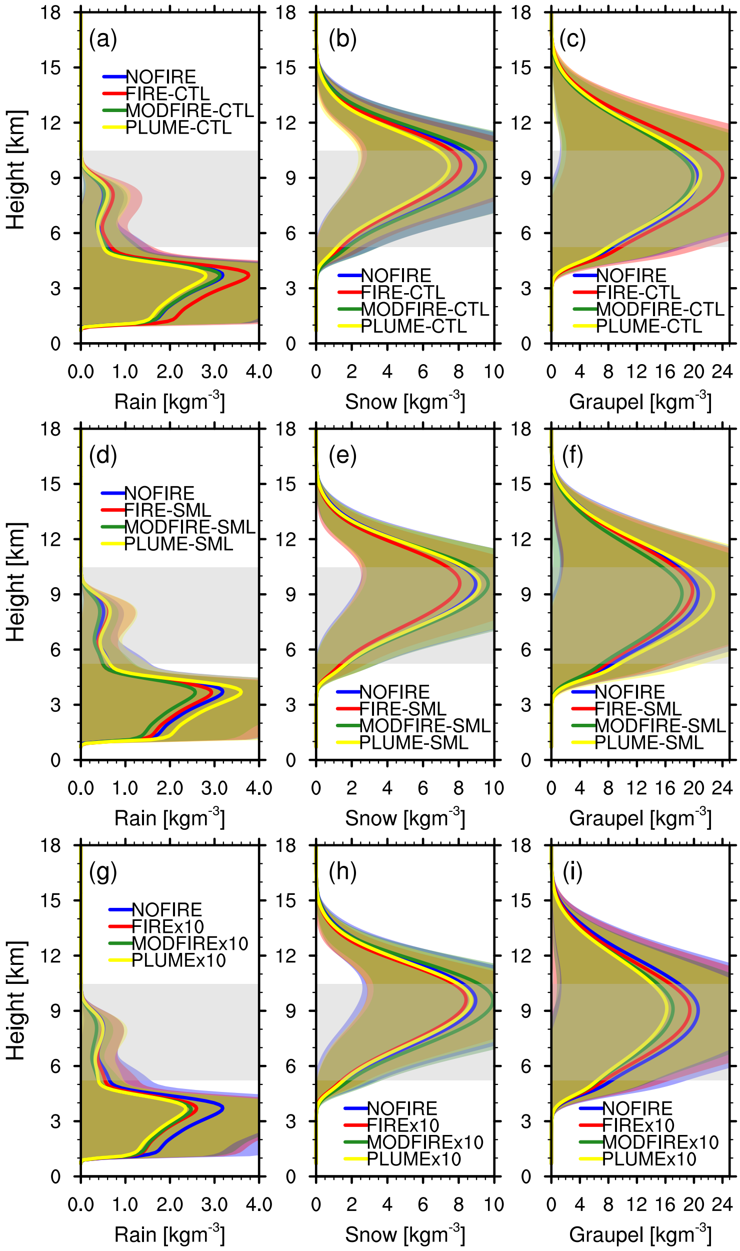

Figure S9. Vertical profiles of horizontally-summed (a,d,g) rain, (b,e,h) snow, and (c,f,i) graupel mass concentrations [kgm^{-3}] in the (a-c) CTL, (d-f) SML, and (g-i) x10 runs, averaged between 20 UTC on 22 June and 02 UTC on 23 June (data every 10 minutes). The shadings show $\pm$ temporal standard deviation. The grey shading indicates the temporally and spatially averaged mixed-phase temperature range in the NOFIRE run. These profiles were vertically interpolated at every 100 m.

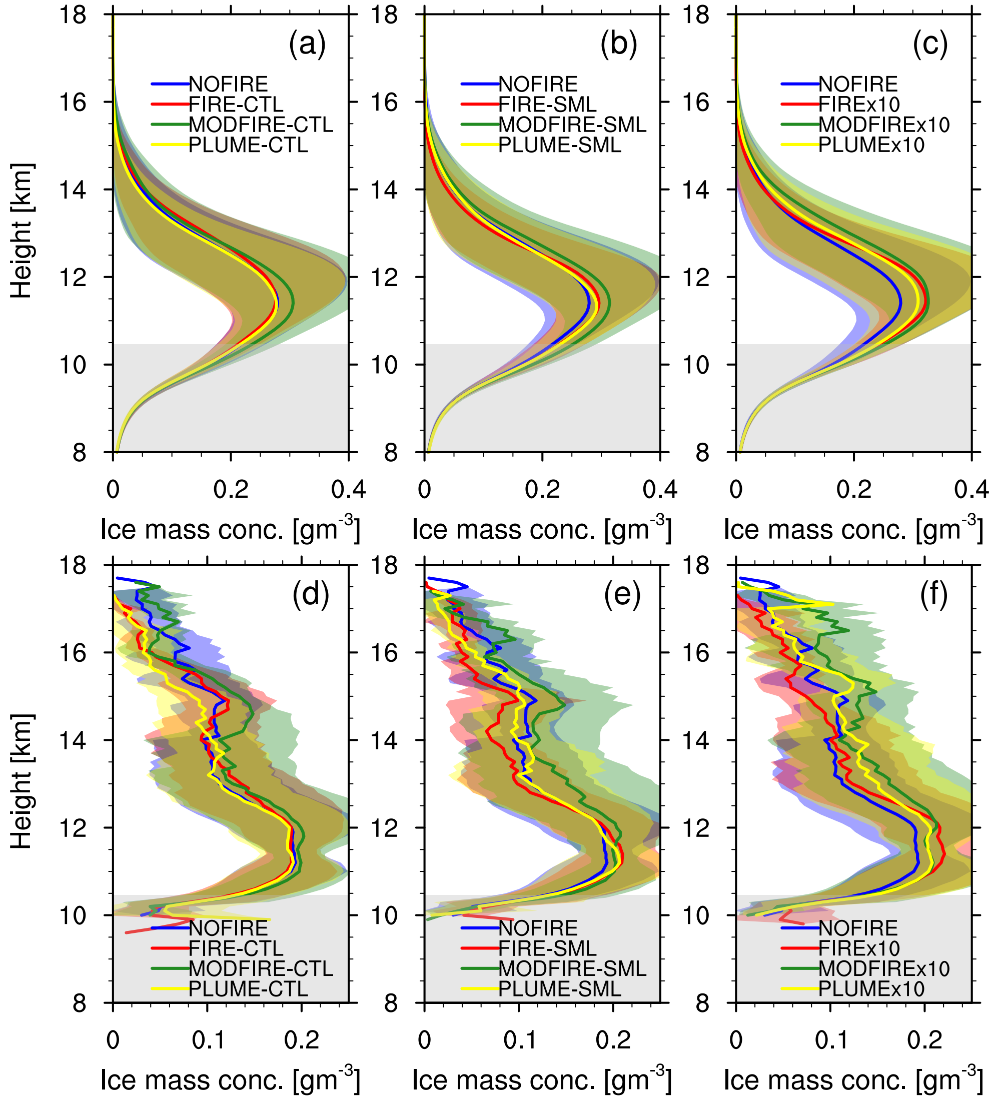

Figure S10. Vertical profiles of ice mass concentrations [gm^{-3}] averaged inside (a-c) columns with one or more convective-core grid boxes and (d-f) anvil clouds between 20 UTC and 2230 UTC on 22 June (data every 10 minutes) in the (a,d) CTL, (b,e) SML, and (c,f) x10 runs. The shadings show ± temporal standard deviation. The grey shading indicates the temporally and spatially averaged mixed-phase temperature range inside convective cores in the NOFIRE run. These profiles were vertically interpolated at every 100 m.

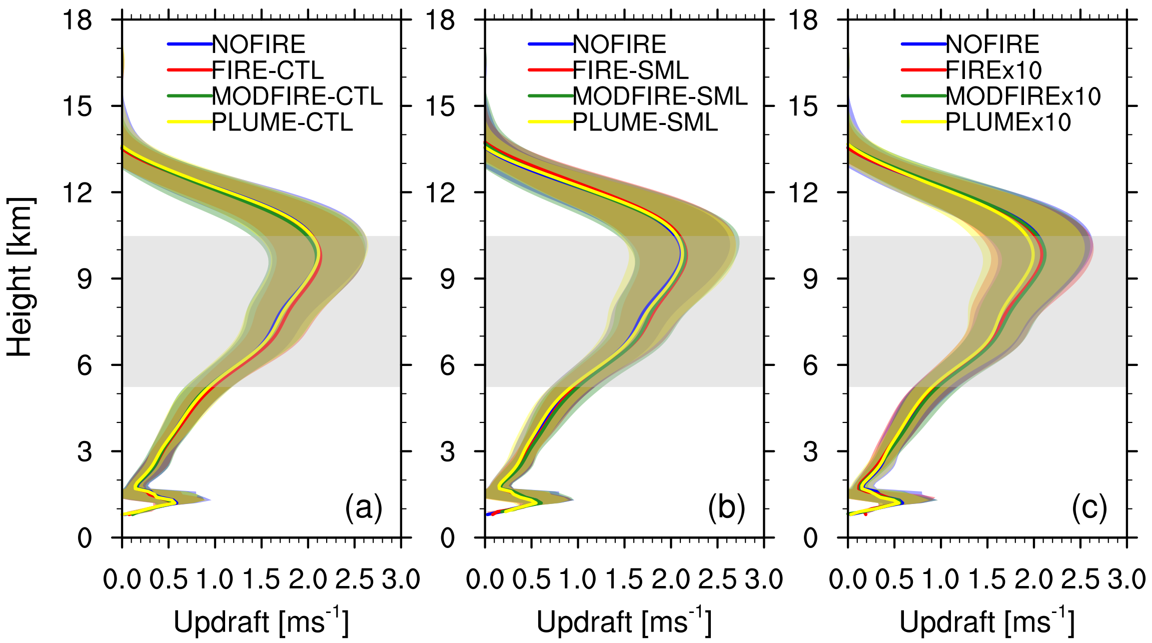

Figure S11. Mean updraft speed [ms^{-1}] inside columns with one or more convective-core grid boxes in the (a) CTL, (b) SML, and (c) x10 runs, averaged between 2000 and 2230 UTC on 22 June (data every 10 minutes). The shadings show ± temporal standard deviation. The grey shading indicates the temporally and spatially averaged mixed-phase temperature range inside convective cores in the NOFIRE run. These profiles were vertically interpolated at every 100 m.

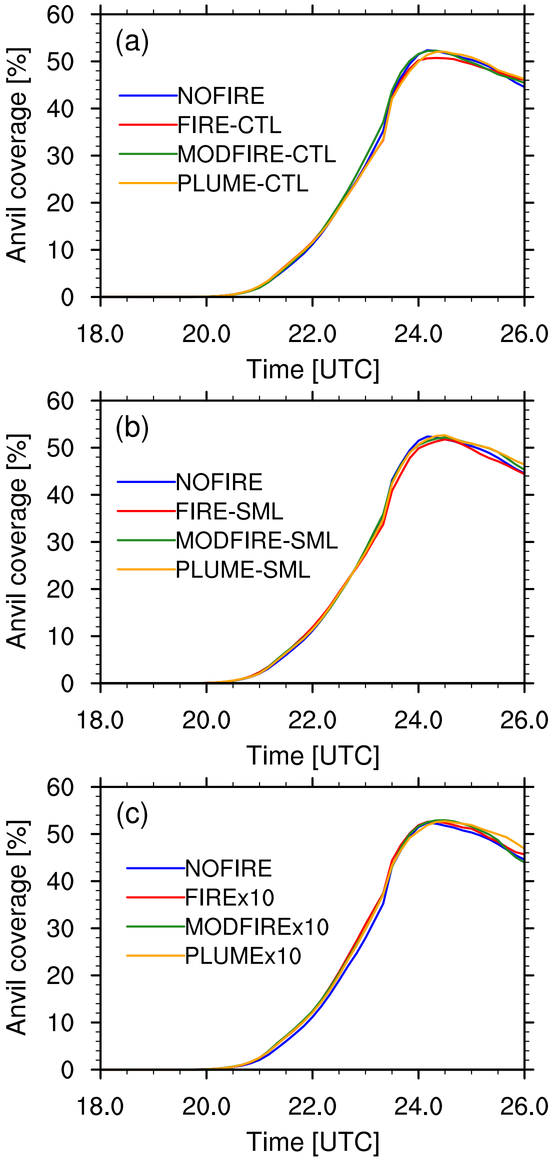

Figure S12. Time series of the percentages [%] of columns with one or more anvil grid boxes in the nested domain in the (a) CTL, (b) SML, and (c) x10 runs.

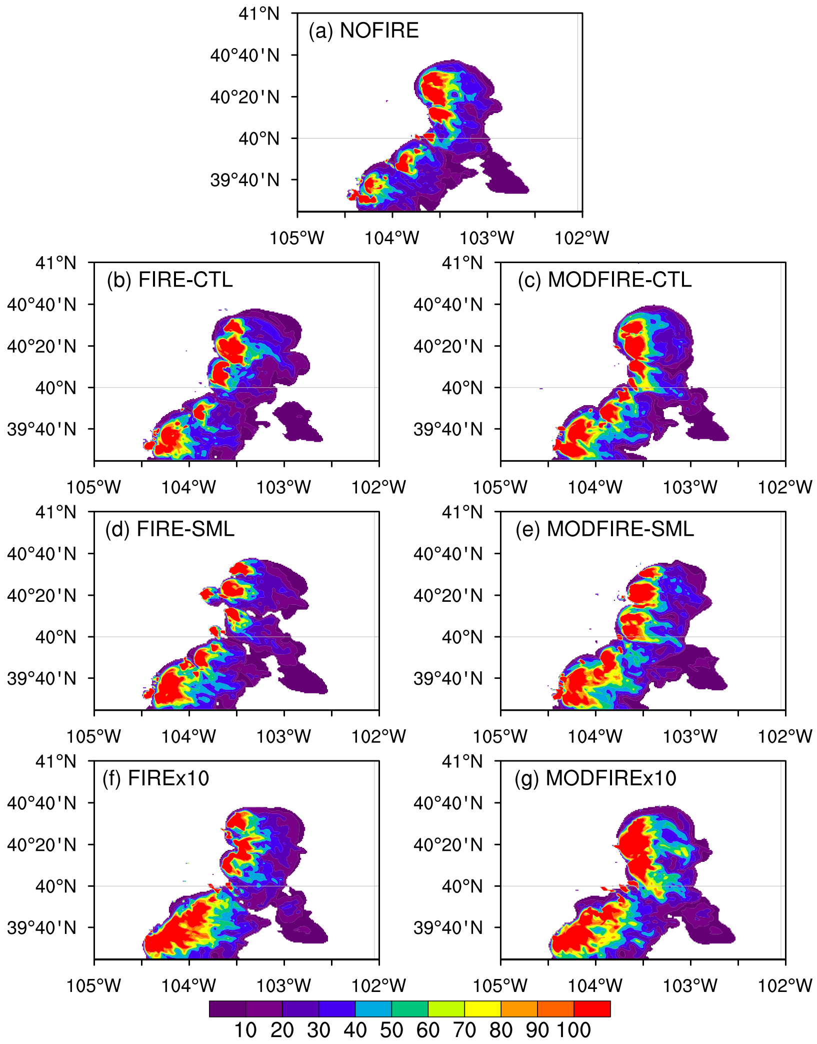

Figure S13. A top-down view of cloud optical depth in (a) NOFIRE, (b) FIRE-CTL, (c) MODFIRE-CTL, (d) FIRE-SML, (e) MODFIRE-SML, (f) FIREx10, and (g) MODFIREx10 at 2140 UTC on 22 June, approximately when the differences in cloud optical depth maximize (Figure 16). These were estimated from the model output of cloud droplet and cloud ice mass and sizes. The computation was done only in grid boxes with either convective cores or anvils.

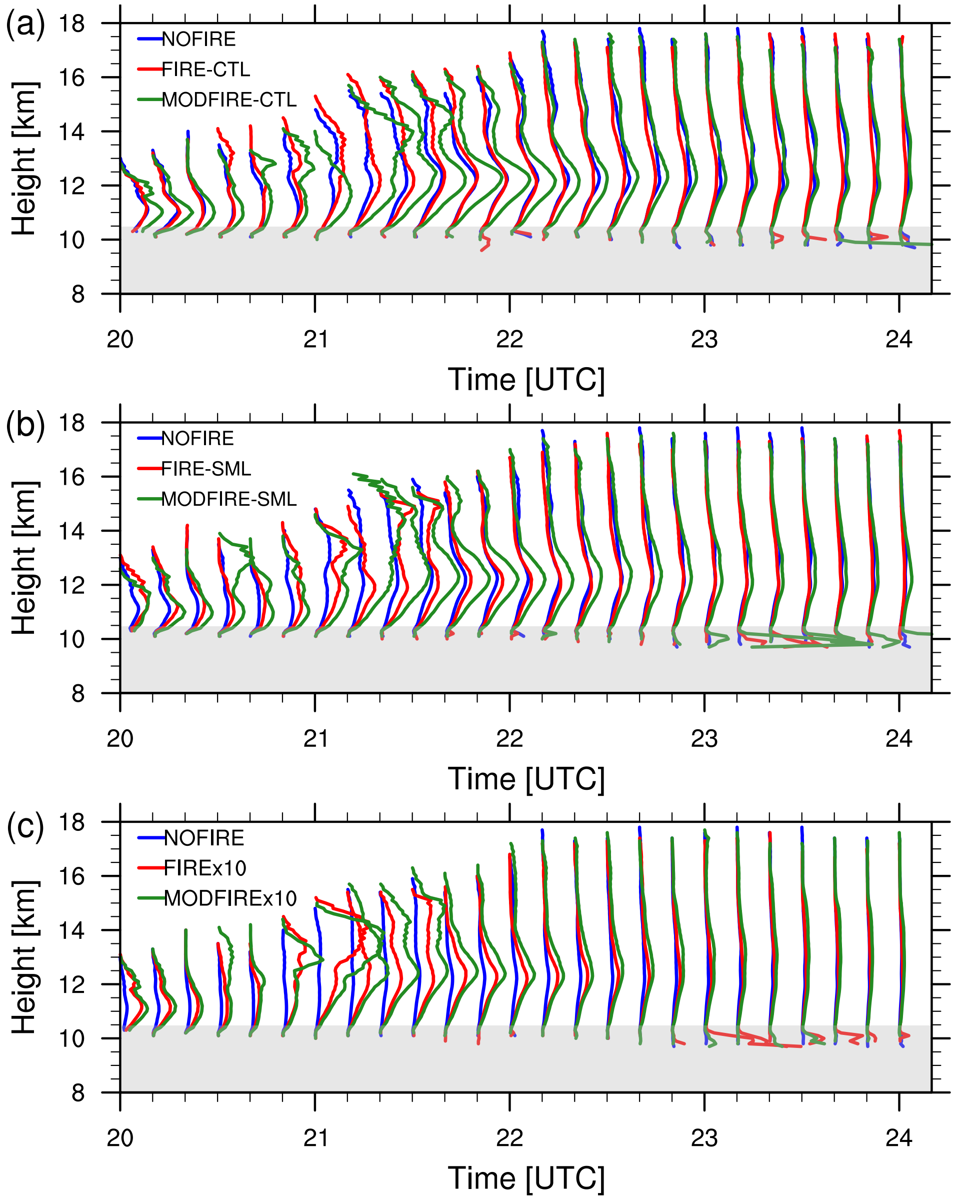

Figure S14. Changes in the vertical profiles of anvil-mean ice crystal number concentrations over time, from 20 UTC on 22 June to 00 UTC on 23 June in the (a) CTL, (b) SML, and (c) x10 runs. Since this figure focuses on the time series of vertical profiles and not the actual concentrations, different sets of simulations (e.g., CTL and SML) were normalized by different values. The grey shading indicates the temporally and spatially averaged mixed-phase temperature range inside convective cores in the NOFIRE run. These profiles were vertically interpolated at every 100 m.

Data Set S1. A zip file that contains namelist and modified module files for the WRF-CHEM simulations conducted in this study (modifications marked by "TAKEISHI");

- ***namelist.wps***: Namelist file for the WRF Preprocessing System (WPS)
- ***namelist.input.NOFIRE***: Namelist file for the NOFIRE simulations
- ***namelist.input.FIRE***: Namelist file for the FIRE, MODFIRE, and PLUME simulations
- ***module_data_sorgam_NAME.F*** (*"NAME"* corresponds to the name of the run in Table 1): Module files for the simulations in which hygroscopicity and emitted particle sizes (the latter only for the SML runs) were modified
- ***module_data_sorgam_116.F***: Module file for the test run in which $\kappa = 1.16$
- ***module_mixactivate_PLUME.F***: Module file for the PLUME runs in which droplet activation was enhanced between 31st and 35th levels
- ***module_mixactivate_high-fraction.F***: Module file for the test simulation in which the fractions of activation were set to 0.25 (Aitken mode) and 1 (accumulation and coarse modes)
- ***module_mixactivate_larger.F***: Module file for the test run in which the sizes of aerosol particles were artificially raised to 25 nm (Aitken), 250 nm (accumulation), and 2.5 μm (coarse) in the calculation of droplet activation
- The other files: Module files modified in order to output additional variables
